# Quantifying nanodiamonds biodistribution in whole cells with correlative iono-nanoscopy

**DOI:** 10.1038/s41467-021-25004-9

**Published:** 2021-08-02

**Authors:** Zhaohong Mi, Ce-Belle Chen, Hong Qi Tan, Yanxin Dou, Chengyuan Yang, Shuvan Prashant Turaga, Minqin Ren, Saumitra K. Vajandar, Gin Hao Yuen, Thomas Osipowicz, Frank Watt, Andrew A. Bettiol

**Affiliations:** 1 Centre for Ion Beam Applications, Department of Physics, National University of Singapore, Singapore, Singapore; 2 Division of Science, Yale-NUS College, Singapore, Singapore; 3 Present Address: Division of Radiation Oncology, National Cancer Centre Singapore, Singapore, Singapore

**Keywords:** Fluorescence imaging, Nanoparticles, Microscopy, Imaging techniques

## Abstract

Correlative imaging and quantification of intracellular nanoparticles with the underlying ultrastructure is crucial for understanding cell-nanoparticle interactions in biological research. However, correlative nanoscale imaging of whole cells still remains a daunting challenge. Here, we report a straightforward nanoscopic approach for whole-cell correlative imaging, by simultaneous ionoluminescence and ultrastructure mapping implemented with a highly focused beam of alpha particles. We demonstrate that fluorescent nanodiamonds exhibit fast, ultrabright and stable emission upon excitation by alpha particles. Thus, by using fluorescent nanodiamonds as imaging probes, our approach enables quantification and correlative localization of single nanodiamonds within a whole cell at sub-30 nm resolution. As an application example, we show that our approach, together with Monte Carlo simulations and radiobiological experiments, can be employed to provide unique insights into the mechanisms of nanodiamond radiosensitization at the single whole-cell level. These findings may benefit clinical studies of radio-enhancement effects by nanoparticles in charged-particle cancer therapy.

## Abstract (web summary)

The authors demonstrate efficient excitation of nanodiamonds by a focused beam of helium ions, resulting in ionoluminescence. They use this for quantification and correlative localization of single particles within a whole cell at sub-30 nm resolution, and investigate nanodiamond radiosensitisation effects.

## Introduction

Nanoscale imaging of whole cells is essential to observe cellular functions, interactions, and dynamics in the native state of the cells. To this end, super-resolution fluorescence techniques, such as stimulated emission depletion microscopy^1^, have become enabling tools to image whole cells of labeled constituents^2–4^. To link the labeled cellular components with the underlying ultrastructure, the super-resolution optical methods have been combined with electron microscopy to provide correlative light electron imaging capabilities^5–7^. However, electrons essentially suffer from significant scattering when interacting with biological samples as thick as a whole cell, which in turn compromises the resolution^8^. As a result, correlative super-resolution imaging of whole cells remains difficult.

In contrast to electrons, hard X-rays and mega-electron-volt (MeV) ions, such as helium ions, offer a significant advantage of being able to penetrate a whole cell with little broadening of the beam spot^8,9^. Fluorescence microscopic techniques have been previously combined with X-ray microscopy or ion beam analysis techniques to perform high-resolution correlative imaging^10,11^. However, these techniques are based on using dual beams to generate images independently and rely on post-image alignment, which might degrade the correlation accuracy. Also, traditional ion beam analysis techniques have been limited by the beam spot sizes achievable. In our study, the beam spot size of MeV helium ions can be focused down to sub-30 nm (see Supplementary Information). As such, the sub-30 nm spatial resolution can be maintained throughout the cell for whole-cell imaging, in stark contrast with electron microscopy. Moreover, a highly focused beam of MeV helium ions allows imaging of cell structure by measuring ion energy loss in the scanning transmission-ion mode^9,12^, while simultaneously performing luminescence imaging by collecting luminescent photons upon excitation by helium ions^13^, at super resolutions. Therefore, we hypothesize that a correlative iono-nanoscopic approach can be implemented for whole-cell luminescence and ultrastructure mapping in a single imaging experiment.

However, MeV ions can be destructive to conventional fluorescent probes (organic dyes, fluorescent proteins, quantum dots, and so on)^12,14^. Recently, fluorescent nanodiamonds, particularly those engineered with nitrogen-vacancy (NV) color centers^15^, have gained increasing attention for use as biomarkers in biological and biomedical imaging^16–19^, and as magnetometers in nanoscale quantum biosensing^20–23^. The increased interest in nanodiamonds can be attributed to their biocompatibility, sustained fluorescence, consistent photostability, and prolonged coherence time of the NV centers^24^. In addition, fluorescent nanodiamonds hosting NV centers can emit in the far-red spectral domain, at a wavelength which is out of the range of most autofluorescent components of cells^12,25^. Therefore, nanodiamonds with NV centers could be ideal fluorescent probes for ion beam imaging of cells.

The stimulation of NV centers has been implemented optically^15,26,27^, electrically^28,29^, and also in the form of cathodoluminescence^30,31^. Here, we report the efficient excitation of NV centers in nanodiamonds by a focused beam of MeV helium ions, which results in photon emission in the form of ionoluminescence. By using nanodiamonds as imaging probes, we demonstrate a correlative iono-nanoscopic approach for ionoluminescence and ultrastructure imaging in whole cells. We further demonstrate that using this approach, we are able to locate and quantify nanodiamonds in single cells at high spatial resolutions. This approach provides important input data when combined with Monte Carlo simulations to evaluate the mechanisms of nanoparticle radiosensitization in cancer treatment such as proton cancer therapy. Here, we show that internalized nanodiamonds in live cells irradiated with protons are unlikely to affect nuclear DNA through the radiosensitizing process of either proton-induced secondary electrons or hydroxyl radicals. This result elucidates a long-standing problem on nanoparticle radiosensitization, where nuclear DNA damage is considered to be the prime mechanism.

## Results

### Instrumentation and implementation of ionoluminescence nanoscopy

**Fig. 1:**
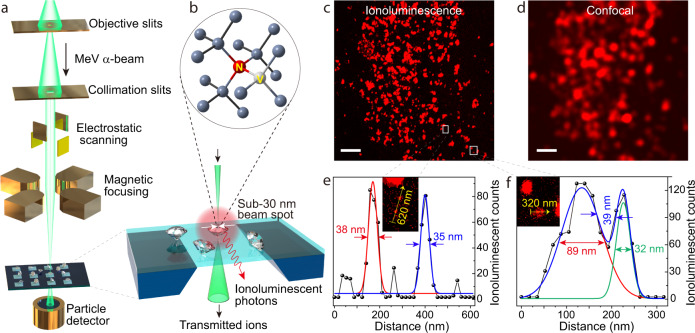
Ionoluminescence nanoscopy. **a** Schematic illustration of the basic beam optics and experimental setup. Note that ionoluminescent photons emitted from the nanodiamonds induced by the α-beam are collected with a parabolic-mirror-based system and detected with a photomultiplier tube for ionoluminescence mapping (see Supplementary Fig. 3). **b** Nitrogen-vacancy (NV) center in the diamond crystalline structure. **c** Ionoluminescence image of the nanodiamonds through α-particle excitation. Scale bar, 2 μm. **d** Confocal image of the same region of the nanodiamond sample taken by using 543-nm laser excitation. Scale bar, 2 μm. **e** Cross-sectional line profile extracted along the arrow shown in the inset of a high-magnification image that corresponds to the region of interest marked in **c**, demonstrating the discrimination of two single nanodiamonds with a size of 38 and 35 nm, respectively. **f** Cross-sectional line profile extracted along the arrow depicted in the inset of a high-magnification image that hosts two single nanodiamonds with a separation distance approximating the resolving limit, directly indicating a sub-40 nm resolution of the ionoluminescence image.

In our approach, a highly focused beam of helium ions (1.6 MeV α-particles) produced with an ion accelerator was instrumental in generating ionoluminescent photons in nanodiamonds (Fig. 1a). The primary beam spot sizes are defined by a set of objective slits, followed by the beam passing through another set of collimating slits, aiming to limit the beam aberrations. The α-beam is then electrostatically raster-scanned before being de-magnified by a magnetic-lens-based focusing system (Supplementary Fig. 1 and Note 1). The beam spot sizes achieved in this work are 22 nm horizontally and 27 nm vertically (Supplementary Fig. 2 and Note 2). A sample comprising fluorescent nanodiamonds prepared on a silicon nitride membrane (100-nm-thick) was positioned precisely at the focal plane of the α-beam. Under excitation by α-particles, NV centers in the nanodiamonds (structure shown in Fig. 1b) emit ionoluminescent photons, which are captured by either a photomultiplier tube for ionoluminescence mapping or a spectrometer for spectroscopic characterization. Concurrently, the energy loss of the transmitted α-particles, which is proportional to the areal density of the sample^12^, was measured by a silicon surface barrier detector to generate density maps of the sample (Supplementary Fig. 3).

To demonstrate the capability of ionoluminescence nanoscopy, an image of the nanodiamonds was acquired by recording the ionoluminescent photon counts in a 1024 × 1024-pixel array (Fig. 1c), at an α-particle count rate of around 12,000 per second (beam current ~2 fA). A representative cross-sectional line profile of two single nanodiamonds was collected and fitted with a modified Gaussian function (Fig. 1e and Supplementary Note 2). This result suggests a sub-40 nm resolution of the ionoluminescence image, which is further confirmed by measuring an area containing two nanodiamonds with a separation distance approximating the resolving limit (Fig. 1f). By comparison, an optical confocal image of the same area of the nanodiamond sample was also acquired (Fig. 1d), and as expected this image exhibits much reduced spatial resolution due to optical diffraction.

### Mechanistic study of ionoluminescence in nanodiamonds

**Fig. 2:**
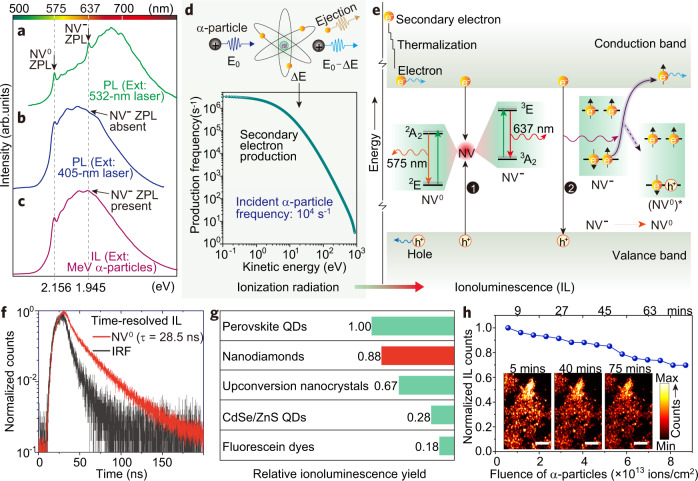
Mechanistic investigation and characterization of ionoluminescence in nanodiamonds. **a** Photoluminescence (PL) spectrum of nanodiamonds excited with a 532-nm laser. **b** Photoluminescence spectrum of nanodiamonds excited with a 405-nm laser. **c** Ionoluminescence (IL) spectrum of nanodiamonds excited with a beam of 1.6 MeV α-particles. **d** Illustration of α-particle-induced atomic ionization in producing secondary electrons (top), through energy deposition (Δ*E*) of the bombarding α-particles (energy of *E*_0_), and calculated energy distribution of the secondary electrons (bottom) in a nanodiamond. **e** Proposed mechanism of ionoluminescence through α-particle excitation. Process 1 represents NV-defect-assisted recombination which results in the emission of NV^0^ and NV¯. Process 2 represents interband recombination which results in the conversion of NV¯ to NV^0^ through ground-state ionization of NV¯, forming NV^0^ in its excited state of (NV^0^)*. **f** Time-resolved ionoluminescence measurement. Note that the instrumental response function (IRF) was determined by measuring the ionoluminescence response of a fast-decay material (Supplementary Fig. 5 and Note 3). **g** Relative ionoluminescence yield measurement. Note that the ionoluminescence yields of nanodiamonds, upconversion nanocrystals (NaYF_4_: Yb/Tm), CdSe/ZnS quantum dots (QDs), and fluorescein dyes (FITC-1907) were normalized with a perovskite-QDs scintillator (Supplementary Figs. 6 and 7 and Note 4). **h** Ionoluminescence intensity profile as a function of the accumulated fluence of α-particles, showing a considerable iono-bleaching resistance of the nanodiamonds. The inserted images, taken at different time intervals (5, 40, and 75 min), indicate that the emission brightness of the nanodiamonds remains essentially unaltered over time. Scale bars are 2 μm.

We next investigated the mechanistic processes that govern ionoluminescent photon emission upon α-particle excitation. We first performed spectroscopic photoluminescence measurement of the nanodiamonds under excitation by a 532-nm laser (Fig. 2a). The nanodiamonds exhibit characteristic emission of sharp zero-phonon lines (575 nm for neutral NV^0^ and 637 nm for negatively charged NV^−^) and their associated phonon sidebands upon 532-nm excitation, confirming that the nanodiamonds host both NV^0^ and NV^−^ emitting centers. In contrast, a 405-nm laser can only excite NV^0^ (Fig. 2b). This is in line with the established fact that high-energy photons (energy above 2.76 eV) are beyond the absorption band of NV^−^ centers^26,27^. Intriguingly, when MeV α-particles (with 10^5^ times the energy of 405-nm photons) are used as excitation sources, both NV^0^ and NV^−^ centers are turned on, resulting in characteristic ionoluminescence emission (Fig. 2c). We thus speculate that α-particle excitation combines the excitation regimes of both low- and high-energy photons.

Figure 2d, e presents a proposed mechanism to elucidate the origin of ionoluminescence emission in NV centers on α-particle excitation. Other than direct energy-level transition with optical photon excitation, the interaction of MeV α-particles with atomic electrons predominantly leads to inelastic energy loss through an atomic ionization process, resulting in the ejection of secondary electrons (Fig. 2d). We carried out kinetic simulations on the production cross-sections of the ionized secondary electrons using a Hansen–Kocbach–Stolterfoht theoretical model^8^. The calculation result shows that energetic secondary electrons (0.1–870.8 eV) can be induced by the primary α-particles (Fig. 2d). As a result, hot holes are generated in the valance band of the nanodiamond. The hot electrons and holes are then rapidly thermalized to the edges of their corresponding occupying bands (Fig. 2e). These electron–hole pairs, carrying kinetic energies that match the absorption bands of NV^0^ or NV^−^, may travel up to the NV emission centers, where indirect carrier recombination through a Shockley–Read–Hall process will dominate^29^. As a result, both NV^0^ and NV^−^ centers emit characteristically through this NV-defect-assisted recombination process.

It should be noted that a radiative band-to-band recombination process, in which the interband energy release (~5.47 eV) corresponds to a wavelength of 227 nm, could also occur (Fig. 2e). The released photons could excite NV^0^ but are unlikely to excite NV^−^ directly since the lower absorption edge of NV^−^ is located in the blue region of the spectrum^26,32^. Notably, however, the energy released from this radiative recombination is sufficient to ionize the negatively charged defects, thereby converting NV^−^ to NV^0^, similar to that of far-UV excitation^32^. And this could be evidenced by the weakened emission of NV^−^ centers whose phonon sidebands are suppressed under α-particle excitation, compared with the distinct phonon sidebands of NV^0^ centers (Fig. 2c).

We took a further step and measured the ionoluminescence properties of the nanodiamonds. Upon excitation with a pulsed beam of α-particles, these nanodiamonds exhibit a very fast response with an ionoluminescence decay time of 28.5 ns (Fig. 2f and Supplementary Fig. 4). The fast response makes it possible to avoid overlapped counting of photons when performing fast ionoluminescence imaging. Also, the nanodiamonds present a superior performance regarding the relative ionoluminescence yield, as compared with lanthanide-doped nanocrystals^13^, semiconducting quantum dots, and organic dyes that are typically employed for bioimaging (Fig. 2g). Moreover, we observed a pronounced resistance of these nanodiamonds to iono-bleaching (Fig. 2h), a phenomenon associated with the reduction in emission intensity with accumulated ion fluence. Note that the typical α-particle fluence required for ionoluminescence imaging in our study was no more than 1 × 10^12^ cm^−2^. Here, to assess the performance of the nanodiamonds under long-term irradiation, we increased the fluence accumulated up to 9 × 10^13^ cm^−2^, which is about two orders of magnitude higher than that needed for performing high-quality ionoluminescence imaging. In addition, it should be mentioned that the creation of new color centers (e.g. NVN) was not observed at an α-particle fluence up to 9 × 10^13^ cm^−2^. The remarkable brightness and iono-stability suggest that nanodiamonds are ideally suited for ionoluminescence imaging under harsh α-particle irradiation.

### Correlative and quantitative iono-nanoscopy of whole cells

**Fig. 3:**
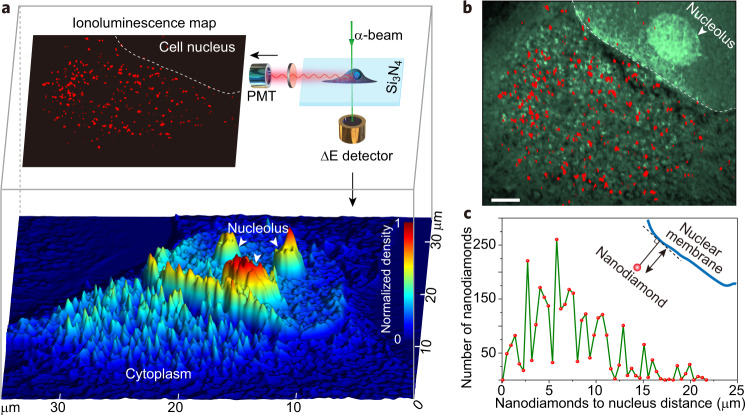
Correlative ultrastructure and ionoluminescence mapping towards quantitative localization of single nanodiamonds in a whole HeLa cell. **a** Demonstration of correlative ultrastructure and ionoluminescence imaging of the HeLa cell with endocytosed nanodiamonds, realized in a single imaging experiment. The basic experimental design (top right) enables simultaneous acquisition of luminescence image of the nanodiamonds and 3D rendering of cell ultrastructure, by capturing α-particle-induced photons with a photomultiplier tube (PMT) and by detecting the energy loss (Δ*E*) of the transmitted α-particles through a Si surface barrier detector, respectively. **b** Overlay of the structural and luminescent images presented in **a**, showing localization of nanodiamonds in the cell. Scale bar, 2 μm. **c** Measurement of the number of intracellular nanodiamonds displayed in **b** in terms of their distance to the cell nuclear boundary.

The mechanistic understanding of ionoluminescence facilitates our subsequent investigations into whole-cell correlative imaging of nanodiamonds by using a focused α-beam. As a proof-of-concept demonstration, HeLa cells incubated with the nanodiamonds were grown onto a 100-nm-thick silicon nitride membrane. A cell of interest was then concurrently imaged in ionoluminescence mode and transmission-ion mode sorted with dual digital channels, by recording the counts of ionoluminescent photons and energy loss of those transmitted α-particles pixel by pixel, respectively. As a result, a luminescence map that shows the biodistribution of the nanodiamonds and a density map that illustrates cellular structures can be simultaneously obtained (Fig. 3a and Supplementary Fig. 8). This approach thereby robustly validates whole-cell correlative localization of intracellular nanodiamonds when the ionoluminescnece map and the density map are overlaid (Fig. 3b). More importantly, the single-particle discrimination capability of ionoluminescence nanoscopy, together with the development of a statistical counting method based on Poisson distribution (see “Methods”), enables quantitative imaging of single nanodiamonds within the cell. For illustration, we have been able to measure the number of single nanodiamonds in terms of their distance to the nuclear boundary within the whole cell (Fig. 3c). Furthermore, it is worth mentioning that our approach could be extended for imaging samples in an atmospheric environment (e.g. living cells), technically similar to liquid cell transmission electron microscopy^6,33^ (see Supplementary Note 5).

### Mechanistic investigation of nanodiamond radiosensitization effects

**Fig. 4:**
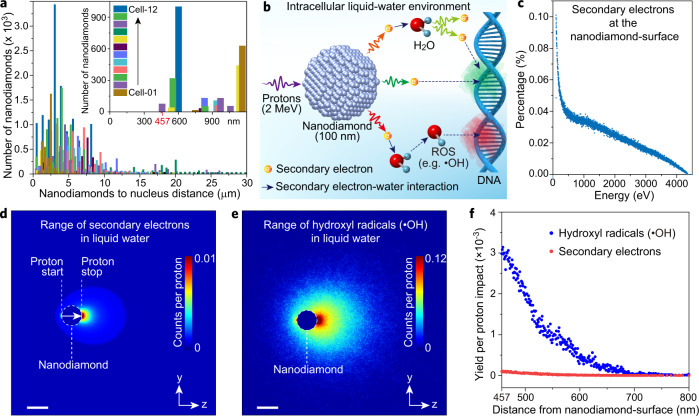
Evaluation of nanodiamonds as proton radiosensitizers. **a** Histogram showing the distribution of nanodiamond-to-nucleus distance that was experimentally measured within 12 HepG2 cells. The magnified inset indicates that zero nanoparticles were found within 457 nm of the nuclear boundary. **b** Plausible mechanisms of nanodiamond-mediated proton radiosensitization in damaging nuclear DNA. Secondary electrons can be induced by the 2-MeV protons when they penetrate a nanodiamond. The secondary electrons that emanate from the nanodiamond, as well as subsequent generations of induced electrons, can either damage nuclear DNA directly or ionize intracellular water molecules in producing reactive oxygen species (ROS) to react with nuclear DNA indirectly. **c** Calculated energy distribution of the secondary electrons escaping the nanodiamond at its surface, simulated with Geant4-DNA. **d** Image showing range distributions of the secondary electrons in liquid water, simulated with Geant4-DNA. Note that we assumed the protons travel only within the nanodiamond (100 nm diameter). Scale bar, 100 nm. **e** Image showing range distributions of hydroxyl radicals (•OH) in liquid water, simulated with Geant4-DNA. Scale bar, 100 nm. **f** Measurement of the number of hydroxyl radicals and secondary electrons per proton impact as a function of their distances from the nanodiamond-surface. Note that only those traveling more than 457 nm (the minimum nanodiamond-to-nucleus distance determined in **a**) were taken into account.

The capability of our approach in achieving whole-cell quantitative localization of nanodiamonds could pave the way to new research areas. For instance, nanodiamonds have been demonstrated in vitro as radiosensitizers to enhance the effect of cancer radiotherapy with γ-irradiation, which had been attributed to the negative electron affinity of the hydrogenated surface of the nanodiamonds^34^. However, other than the surface effect, it was not clear if the intrinsic property of the nanodiamonds could be responsible for the radiosensitization effect. Indeed, more generally the pathways of nanoparticle radiosensitization are still in dispute. The cell nucleus has been considered as the main target for ionizing radiation, and nanoparticles generally do not enter the nucleus (evidenced in Fig. 4a). Such assumptions imply that any radio-enhancement effect is derived from the secondary electrons that emanate from the nanoparticles located in the cytoplasm when interacting with primary radiation. The travel ranges of the secondary electrons and subsequent reaction products can be simulated. However, to quantitatively assess if these secondary products have a range that can affect nuclear DNA, we need to know the nanoparticle-nucleus distance in a whole cell. This is now achievable by using our correlative iono-nanoscopic approach.

In our subsequent studies, we chose to use human hepatoblastoma cells (HepG2) that were established models employed for radiobiological experiments. By using our correlative iono-nanoscopic approach, a total of 12 dried HepG2 cells were imaged and measured to chart the distribution of nanodiamonds-to-nucleus distance (Fig. 4a and Supplementary Figs. 9 and 10). In particular, a minimum distance of 457 nm between the closest nanodiamonds and the nuclei of the HepG2 cells was determined (Fig. 4a).

As an example, we consider here the case of proton irradiation in cancer therapy^35^, and assess the validity of nanodiamonds-mediated radiosensitization mechanisms by using Geant4-DNA Monte Carlo simulations (Fig. 4b). In our model, we assume the nanodiamond is spherical with a diameter of 100 nm. The energy spectrum of proton-induced secondary electrons escaping the nanodiamond at its surface was calculated (Fig. 4c). These secondary electrons were assumed to serve as a source to produce reactive oxygen species when they interact with the cell cytoplasm (simulated with water). Monte Carlo simulations using Geant4-DNA were performed to trace secondary electrons and reactive radicals (the most harmful hydroxyl radical as an example), and their end-of-ranges were recorded (Fig. 4d, e).

Next, we considered the secondary electrons and hydroxyl radicals that could travel more than 457 nm to reach the cell nucleus (Fig. 4f). To deliver a typical clinically relevant dose of 2 Gy in a HepG2 cell, a nanodiamond will experience an impact by 0.06 protons on average (Supplementary Note 6). By integrating the total counts from Fig. 4f, the secondary electrons and the hydroxyl radicals derived from one nanodiamond that can reach the nucleus were determined to be 0.002 and 0.015 per 0.06-proton impact respectively, which is negligibly low. Moreover, hydroxyl radicals react rapidly and are unable to directly penetrate the nuclear membrane^36^. These results quantitatively indicate that neither secondary electrons nor hydroxyl radicals derived from the nanodiamond can affect the nuclear DNA under proton irradiation. It is worth noting that, compared to protons, heavier particles (e.g. helium ions and carbon ions) induce secondary electrons with even lower travel ranges (Supplementary Fig. 11), and are therefore not considered here.

**Fig. 5:**
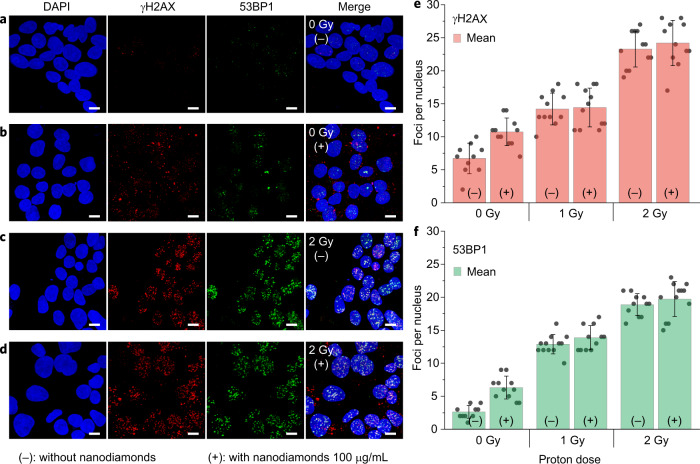
Experimental evaluation of nanodiamond radiosensitization upon proton irradiation of live HepG2 cells. Confocal microscopic images of **a**, unirradiated (0 Gy) control cells without nanodiamonds (−), and **b** unirradiated control cells with 100 μg/ml nanodiamonds (+), in contrast to **c**, irradiated (2 Gy) cells without nanodiamonds, and **d** irradiated (2 Gy) cells with nanodiamonds. Dual-staining for γH2AX (red) and 53BP1 (green) indicates DNA double-strand breaks in the nuclei (blue). Note that the red clusters in the perinuclear regions in **b** and **d** are nanodiamonds. All scale bars in **a**–**d** are 10 μm. **e** Bar graph depicting red foci counts (γH2AX) per nucleus in terms of proton dose (0, 1, and 2 Gy). *n* = 3 biologically independent samples. **f** Bar graph depicting green foci counts (53BP1) per nucleus in terms of proton dose (0, 1, and 2 Gy). *n* = 3 biologically independent samples. Note that in **e** and **f**, each data point represents the measurement of foci counts in one 3D confocal image that contains 10–20 cells. The value of foci per nucleus was obtained by averaging total foci counts with total nuclear volume in the 3D confocal image, and subsequently normalizing with the volume of a typical nucleus (10 × 10 × 10 μm^3^). For each sample, 150–200 cells were analyzed. The data in **e** and **f** are shown as the mean ± s.d. Mann–Whitney test (two-tailed) was used to determine statistical significance. Foci counts between control or cells containing nanodiamonds were not statistically significantly different for γH2AX foci (*p* = 0.94 and *p* = 0.35 at 1 and 2 Gy, respectively) or 53BP1 foci (*p* = 0.28 and *p* = 0.24 at 1 and 2 Gy, respectively).

To validate the simulation results, we further designed experiments to explore DNA double-strand breaks of live HepG2 cells upon irradiation by a broad beam of protons with an effective energy of about 2 MeV (see “Methods”). Two groups of cells were prepared. The first test group of three samples was treated with hydroxylated nanodiamonds, and the second control group of three samples prepared without the nanodiamonds. Two samples from each group were irradiated with 1 or 2 Gy protons, and the other one was unirradiated as a control. After irradiation, phosphorylated histone protein H2AX (γH2AX) and p53-binding protein 1 (53BP1), which are considered to be sensitive markers of DNA double-strand breaks^37,38^, were assayed by observing their foci in cell nuclei (Fig. 5a–d).

For each sample, three-dimensional (3D) confocal microscopic images of 150–200 cells were taken and analyzed to determine the foci counts of γH2AX (Fig. 5e) and 53BP1 (Fig. 5f). As can be seen from Fig. 5e, f, the control cells without nanodiamonds and unirradiated (0 Gy) showed only a few foci per nucleus, whereas the control cells without nanodiamonds but with proton irradiation (1 or 2 Gy) showed an expected significant increase in both γH2AX and 53BP1 foci. Similarly, the test cells containing nanodiamonds and irradiated with protons showed a large increase in foci for both 1 and 2 Gy irradiations, in contrast to those test cells containing nanodiamonds but unirradiated. Interestingly, however, in these irradiated groups, the cells treated with nanodiamonds did not result in an increase of the foci compared to those without nanodiamonds. These results demonstrate that the hydroxylated nanodiamonds employed in our study do not provide radio-enhancement of nuclear double-strand breaks under proton irradiation. This agrees well with our simulation results.

## Discussion

We have demonstrated correlative iono-nanoscopy as a powerful technique to image, localize, and quantify fluorescent nanodiamonds at sub-30 nm resolution throughout a whole cell, by taking advantage of a highly focused MeV α-beam. Our results reveal fast, ultrabright, and stable ionoluminescence emission of fluorescent nanodiamonds on excitation with MeV α-particles. The NV^0^ emitting centers in nanodiamonds were found to dominate the ionoluminescence emission, primarily through NV-defects-assisted recombination. Furthermore, the experimental results of whole-cell correlative localization of nanodiamonds, together with Geant4-DNA Monte Carlo simulations, enable us to shed light on nanodiamond radiosensitization effects at the sub-cellular level. Our findings indicate that the mechanism of nanodiamond-mediated radiosensitization for proton irradiation does not occur through damaging nuclear DNA by induced secondary electrons or hydroxyl radicals. This has been confirmed experimentally by demonstrating that the presence of nanodiamonds in the cytoplasm does not appear to enhance nuclear DNA damage through double-strand breaks upon proton irradiation. Further, we have also countered the argument that the presence of cytoplasmic nanodiamonds significantly enhances DNA damage in the absence of proton irradiation. At the levels of nanodiamond density considered in our study, any enhancement of nuclear DNA double-strand breaks is minimal compared with the effects of proton irradiation.

Our study elucidated a long-standing argument in charged particles-induced radiosensitization of nanoparticles. Though further clinical studies should be performed to validate our results, we believe our findings can deepen the fundamental understanding of nanoparticle-radiosensitization mechanisms to facilitate charged-particle cancer therapy in the era of precision medicine^35,39^. For instance, to enable radiosensitization, nanoparticles could be carefully designed or screened to enrich the emission of electrons^34^. In addition, our unique technique of correlative iono-nanoscopy of whole cells may benefit many other emerging applications, for example, intracellular delivery and screening of potential COVID-19 therapeutics and vaccines^40,41^.

## Methods

### Nanodiamond sample preparation

The hydroxylated nanodiamonds (900 NV centers per particle and positive zeta potential) used in this study were sourced from Adámas Nanotechnologies. The nanodiamonds were diluted to 100 µg/ml, sonicated for 5 min, dropped onto a 100-nm-thick silicon-nitride membrane substrate, and air-dried before being transferred for ionoluminescence nanoscopic imaging and scanning electron microscopic (SEM: JEOL JSM-6700F) analysis. SEM characterizations and size measurement of the nanodiamonds can be found in Supplementary Fig. 12 and Note 7.

### Cell sample preparation for correlative imaging

Human cervical carcinoma cells (HeLa, ATCC) or human hepatoblastoma cells (HepG2, ATCC) were cultured in Dulbecco’s modified Eagle’s medium (DMEM) supplemented with fetal bovine serum (10%), antibiotics penicillin (100 units/ml) and streptomycin (100 µg/ml) at 37 °C in 5% CO_2_ atmosphere. Cells were then seeded onto sterilized silicon-nitride-membrane (100-nm-thick) substrates at an estimated density of 10,000 cells/cm^2^ and allowed to adhere overnight. After attachment, control cells were briefly rinsed with phosphate-buffered saline (PBS) and further incubated with fresh supplemented DMEM for 16 h. Other cells awaiting nanodiamond uptake were incubated similarly but with the addition of nanodiamonds (100 μg/ml) in supplemented DMEM. Cells were then washed thoroughly with PBS prior to fixation in formaldehyde (4%). Fixed cells were intermediately dehydrated by passage through an increasing ethanol gradient, followed by complete dehydration using critical point drying.

### Instrumentation and correlative imaging

Confocal imaging of nanodiamonds was performed by using a laser scanning microscope (Zeiss LSM 510) equipped with a 543 nm helium–neon laser and a ×20 objective lens (numerical aperture: 0.75). In this case, the spatial resolution of imaging was measured to be 186 nm. For ionoluminescence analysis, the MeV α-particles were produced with a single-ended electrostatic particle accelerator (HVEE Singletron^TM^) inbuilt with a radio-frequency ion source. The nanodiamond sample was placed in a vacuum chamber (10^−6^ mbar) at a position situated along the α-beam path at the beam focus. The α-particle-induced photons were collected with a customized double-piece parabolic mirror^42^, and captured either by a photomultiplier tube (Hamamatsu R7400P) for luminescence imaging or by a spectrometer (Ocean Optics QE65000) for spectroscopic characterization with the SpectraSuite software (Ocean Optics). Concurrently, the front and rear openings designed on the parabolic mirror allow the focused α-beam to pass through the sample and exit. An Si surface barrier detector (Ortec) was placed downstream to measure energies and the number of transmitted α-particles after penetrating a given sample. The energy loss, which is proportionally related to the sample’s density at each pixel within the scanned area, was thus determined. When the sample is a cell, the photon counts and energy loss data were collected and processed using the IONDAQ data acquisition system^43^ to generate correlative ionoluminescence and structure maps of the cell, respectively. ImageJ 1.48v was used to generate line profiles from images.

### Time-resolved ionoluminescence

The time-resolved ionoluminescence system was constructed by driving a beam blanker with a high-voltage pulse generator (AVTech AV-1010-B, rise-time 10 ns) to discretize the α-beam, and using the same signal to synchronize with the time-correlated single-photon counting hardware (PicoQuant Timeharp 260 PICO). A hybrid photomultiplier detector (PicoQuant PMA Hybrid 40), coupled with a band-pass filter, was employed to detect single ionoluminescent photons associated with NV^0^ centers in nanodiamonds. A single-photon statistical histogram was formed over multiple cycles by registering photon arrivals per time bin (50 ps) with the software TimeHarp 260 v3.1 (PicoQuant), referenced by using a fast-decay InGaN quantum-well material^44^. The lifetime of the NV^0^ centers was thus determined by fitting the histogram with an exponential decay function.

### Relative ionoluminescence yield measurement

The ionoluminescence yield of a luminescent nanoparticle is related to many factors, such as the nanoparticle size, light collection and detection efficiency, energy deposition and beam-current fluctuation of the incident α-particles. Therefore, it is difficult to determine the absolute ionoluminescence quantum yields of nanoparticles. Instead, we have been able to perform relative ionoluminescence yield measurements. By using the same light collection and detection geometry, we simultaneously took an ionoluminescence image and a scanning transmission ion microscopy^12^ image of each kind of luminescent nanoparticles employed in our study. Thereafter, the number of ionoluminescence counts, the number of incident α-particles and their energy loss at each imaging pixel were acquired from the list-mode data files of these two images. The number of ionoluminescence counts was then divided by the counts and energy loss of α-particles. Finally, a relative ionoluminescence yield was obtained by accumulating the weighted ionoluminescence counts and normalized with a referenced inorganic perovskite quantum-dots scintillator^45^ whose ionoluminescence yield was set to be unitary.

### Nanodiamond counting in single whole cells

We proposed a semiautomatic method for statistical nanoparticle counting in ionoluminescence images of a single whole cell, based on using the technique of image segmentation and Poisson statistics. Our approach primarily depends on the identification of single nanodiamonds and their clusters by differentiating them from the background through the correlation between the ionoluminescence image and the correlated structural image, which is demonstrated in Fig. 3. For each pixel of the ionoluminescence image, we consider *N* ionoluminescent photons are registered by the detector, *N*_p_ nanodiamonds are present at that pixel, the average number of photons emitted per nanodiamond is *λ*, and *λN*_p_ is thus the average number of photon counts contributed by each particular pixel, which we assumed follows Poisson distribution. On the other hand, since the registered photon counts *N* is dependent on the number of nanodiamonds present in the corresponding pixel (*N*_p_), a Poisson distribution assumption for *N*_p_ is also needed to fully capture the statistical property of the photon counts detected. Therefore, a joint probability mass function for *N* and *N*_p_ can be given by a double Poisson model which is presented in Eq. (1):

1 $$\,P(N|{{\lambda }},\beta )=\mathop{\sum }\limits_{{N}_{p}=0}^{\infty }\frac{{e}^{-{{\beta }}}{(\beta )}^{{N}_{p}}}{{N}_{p}!}\frac{{e}^{-{{\lambda }}{N}_{p}}{(\lambda {N}_{p})}^{N}}{{{N}}!}$$

where *β* represents the average number of nanodiamonds in each pixel that was recognized as a part of the identified single nanodiamonds and their clusters, and *λβ* thus equals to the average number of photons detected per pixel occupied by those single nanodiamonds and clusters. Note that in Eq. (1), *N* is the only variable that is experimentally detectable, and the parameters *λ* and *β* are determined by fitting experimental data. If the total number of ionoluminescent counts after background deduction is *C*, the number of pixels containing nanodiamonds is equal to *C*/(λβ). Lastly, if each nanodiamond occupies *A* pixels, the total number of nanodiamonds present in the ionoluminescence image is calculated as *C*/(*Aλβ*). In this step, considering that the size uniformity of nanodiamonds is also crucial to the counting accuracy, we expressed *A* in terms of the size distribution function that is determined in Supplementary Fig. 12. The total number of nanodiamonds in each of the 12 HepG2 cells we measured is shown in Supplementary Table 1. Detailed information on image segmentation is provided in Supplementary Note 8 and Supplementary Fig. 13.

### Monte Carlo simulations of nanodiamond-mediated radiosensitization

Simulations were performed based on using the Geant4-DNA package, which is an extension to the Geant4 (version 10.5) Monte Carlo toolkit^46^. Because the default interaction medium in Geant4-DNA processes is liquid water, in the first step we have chosen G4EmStandardPhysics_option4 constructor that includes general-purpose physics models to describe the interaction of protons with the inside of a nanodiamond. This option allows the tracking of secondary electrons with energy down to 100 eV. In the simulation, we assumed a 100-nm diameter of the nanodiamond, and 1 × 10^7^ protons with an energy of 2 MeV were used to reach a reasonable statistical accuracy. Thereafter, the secondary electrons that escape the nanodiamond at its surface were filed to source the interaction with liquid water outside the nanodiamond, which was simulated by using the G4EmDNAPhysics_option4 constructor that is incorporated in the Geant4-DNA package, allowing tracking of induced electrons with energy down to 10 eV in this case. The step size for all induced electrons was set to 5 nm. Meanwhile, the G4EmDNAChemistry constructor was chosen to simulate and track up to seven chemical species (Supplementary Table 2), within a time domain of 1 µs. The counts of induced secondary electrons and hydroxyl radicals were sorted based on their stop sites into two pixel-arrays, respectively, to indicate their ranges in water by coding with MATLAB. 3D presentations of the travel ranges of the induced secondary electrons and the hydroxyl radicals are provided in Supplementary Fig. 14.

### Live-cell irradiation and immunofluorescence

Monolayer HepG2 cells were seeded on a gelatin-coated Mylar film secured in a custom cell dish and incubated with or without nanodiamonds (16 h) using the same procedure as stated above. A second Mylar film together with a capping ring was mounted to seal the cells prior to proton irradiation^47^. Cells were then irradiated with a homogeneous beam of 2.5 MeV protons. Note that before reaching the cells, the protons traversed successively a beam-exit window (12.5-μm-thick Kapton), an air gap (~5 mm), and a thin substrate (12-μm-thick Mylar for cell adherence). This resulted in an energy loss of the protons, and the effective energy retained for cell irradiation was calculated to be about 2 MeV. Immediately after the irradiation, the cells were incubated for 45 min to allow DNA-damage response proteins to accumulate at the damaged sites. Following aldehyde fixation, the cells were permeabilized with Triton X-100, and blocked with goat serum. Subsequently, the cells were probed with antibodies (1:500) against γH2AX (Merck) or 53BP1 (Novus Biologicals). The signals of γH2AX or 53BP1 were amplified with AlexaFluor 568- or AlexaFluor 488-conjugated secondary antibodies (Thermo Fisher Scientific, 1:500), respectively.

### Confocal imaging and foci counting

The cells were imaged using an LSM 880 laser scanning confocal microscope (Carl Zeiss) through a ×63 oil immersion objective lens (numerical aperture: 1.4), excited with 561 nm or Argon multi-line lasers. For each cell sample, 150–200 cells were imaged 3D. The 3D images were then processed for foci (γH2AX or 53BP1) counting and nuclear volume calculation by coding with Python 3.7. The code is publicly available at https://github.com/CIBA-Physics-NUS/FociApp. The details of image processing and thresholding are provided in Supplementary Note 9.

### Statistics and reproducibility

All the experiments including those in Supplementary Information were performed at least three times independently with similar results.

### Reporting summary

Further information on research design is available in the Nature Research Reporting Summary linked to this article.

## Supplementary information

Supplementary informaition

Reporting Summary

## Data Availability

The datasets that support the findings of this study have been deposited in the Zenodo repository under a Creative Commons Attribution 4.0 International License (Open Access) at 10.5281/zenodo.5068754.

## Code availability

Source files for Geant4 software (version 10.5) can be downloaded at https://geant4.web.cern.ch/node/1765. The Geant4-DNA extension is included in the Geant4 public distribution. The custom codes for image processing and foci counting in confocal images of cells are publicly accessible at https://github.com/CIBA-Physics-NUS/FociApp.
