# Supplementary material for: Quantifying nanodiamonds biodistribution in whole cells with correlative iono-nanoscopy
Source: Nat Commun. 2021 Aug 2;12:4657. doi: 10.1038/s41467-021-25004-9 (PMC8329174; doi:10.1038/s41467-021-25004-9)
Supplement: Supplementary file 1 — Supplementary informaition [file 41467_2021_25004_MOESM1_ESM.pdf]

Supplementary Information

Quantifying nanodiamonds biodistribution in whole cells with correlative iono-nanoscopy

Zhaohong Mi, Ce-Belle Chen, Hong Qi Tan, Yanxin Dou, Chengyuan Yang, Shuvan Prashant Turaga, Minqin Ren, Saumitra K. Vajandar, Gin Hao Yuen, Thomas Osipowicz, Frank Watt, and Andrew A. Bettiol

This Supplementary Information includes Supplementary Notes 1-9, Figs. 1-14, Tables 1-2, and Refs. 1-16, as follows.

Supplementary Note 1. MeV-beam focusing …………………………………………………………… S3
Supplementary Fig. 1 | Experimental setup and principle for MeV beam focusing ……………………… S4
Supplementary Note 2. Beam spot size determination ……………………………………………………… S5
Supplementary Fig. 2 | Beam-spot size measurement of 1.6 MeV focused α-particles …………………... S6
Supplementary Fig. 3 | Schematic illustration of simultaneous density and ionoluminescence mapping …S7
Supplementary Fig. 4 | Time-resolved ionoluminescence …………………………………………………… S8
Supplementary Note 3. Instrumental response function for time-resolved ionoluminescence …………….S9
Supplementary Fig. 5 | InGaN quantum-wells for instrumental response function determination……..…..S10
Supplementary Note 4. Relative ionoluminescence yield measurement ……………………………..........S11
Supplementary Fig. 6 | Ionoluminescence of the $CsPbBr_3$ quantum-dots scintillator ……………….........S11
Supplementary Fig. 7 | Raw images of the luminescent nanomaterials under study ……………………....S12
Supplementary Fig. 8 | Single whole HeLa cell imaging……………………………………………………S13
Supplementary Note 5. Possibility of live-cell ion beam imaging ……………………………………..…..S14
Supplementary Fig. 9 | Single whole HepG2 cell imaging…………………………………………………S15
Supplementary Fig. 10 | Quantitative measurement of the nanodiamonds bio-distribution in HepG2 cells..S16
Supplementary Note 6. Simulation considerations based on a clinically relevant dose……………………S17
Supplementary Table 1. Number of nanodiamonds in individual HepG2 cells……………………………S17
Supplementary Fig. 11 | Range distributions of the secondary electrons derived from the nanodiamond with the impact by different ion species……………………………………………………………………………S18
Supplementary Note 7. Measurement of the size of nanodiamonds ………………………………………S19
Supplementary Fig. 12 | Scanning electron microscopic analysis ……………………………………….....S19

Supplementary Note 8. Image segmentation for nanodiamond counting in cells …………………….....S20
Supplementary Fig. 13 | Algorithm chain of ionoluminescence image segmentation ……………………..S20
Supplementary Table 2. Travel-range simulations of 7 chemical species by using Geant4-DNA…………S21
Supplementary Fig. 14 | Geant4-DNA Monte Carlo simulations …………………………….………S21
Supplementary Note 9. Foci counting in 3D confocal microscopic images …………………………....S22
Supplementary References ………………………………………………………………………....S23

Supplementary Note 1. MeV-beam focusing.

The MeV-beam focusing is achieved with magnetic quadrupole lenses (Supplementary Fig. 1a). As sketched in Supplementary Fig. 1b, a single quadrupole lens tends to converge the beam along one direction, whilst diverging the beam of the other direction. To focus the beam down to a tiny spot, at least two quadrupole lenses should be used. To achieve the sub-30 nm focusing performance, we have employed a single-spaced Oxford triplet configuration of the lenses[1], where there is one-lens space between the first and the second lenses, as shown in Supplementary Fig. 1c. Note that the first and the second lenses are coupled, i.e. fed in series with the same current via one power supply.

Such an arrangement of the lenses enables large de-magnifications to be achieved in both vertical and horizontal focusing planes. The de-magnifications of the single-spaced quadrupole triplet are 574 horizontally and 93 vertically. In our study, the beam brightness is about 22 pA/($\mu m^2 \cdot mrad^2 \cdot MeV$) for 1.6 MeV alpha particles. In the effort to achieve sub-30 nm imaging capability, typical horizontal and vertical openings of the objective slits are 5 μm and 1 μm respectively, and the openings of the collimation slits are 30 μm and 20 μm respectively. The resulting count rate of the α-particles is around 12000 per second.

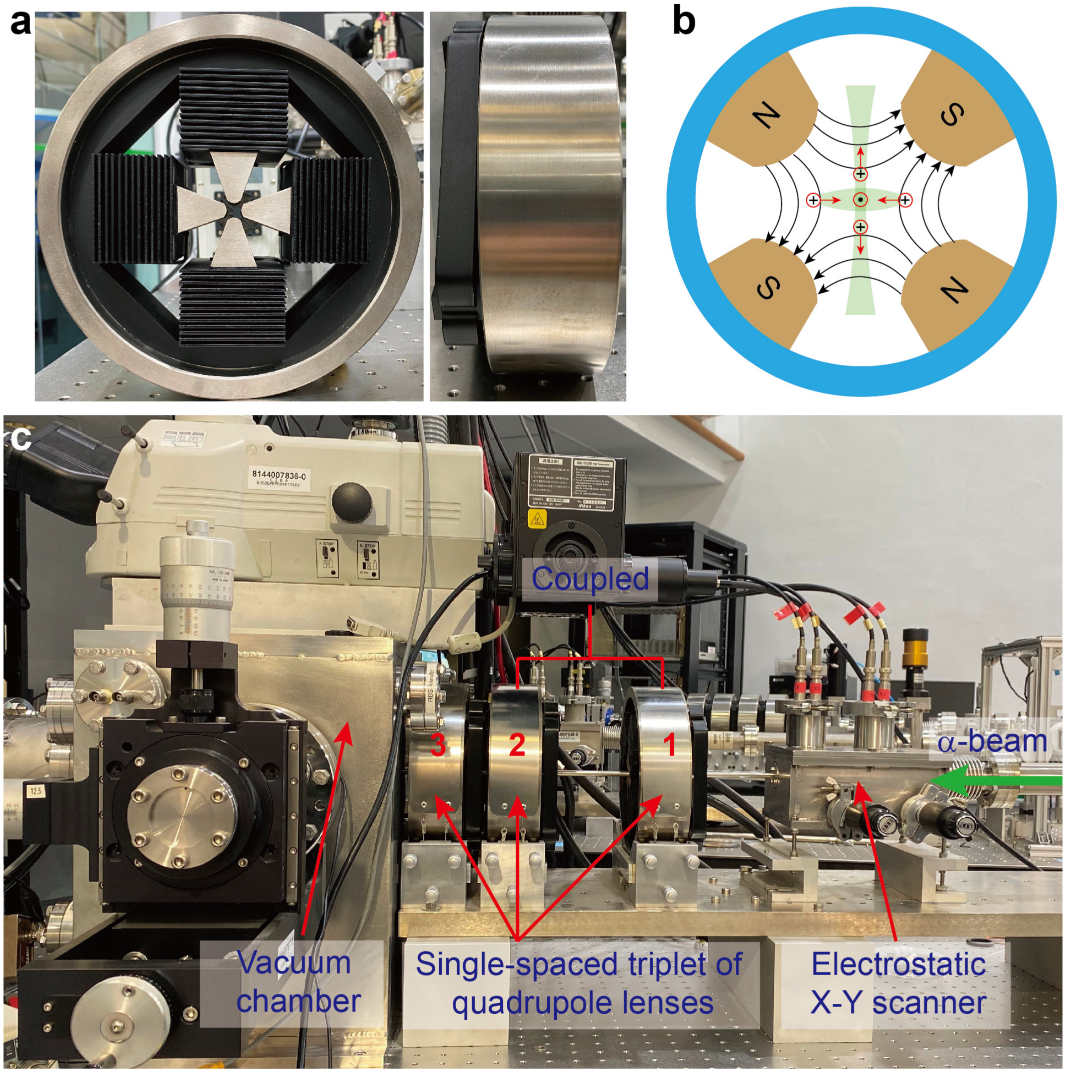

Supplementary Fig. 1 | Experimental setup and principle for MeV ion beam focusing. a, Image showing a magnetic quadrupole lens (front and side views) employed in our study. **b**, Schematic presentation of the beam focusing principle. Note that the positive ions are assumed to travel out of the paper perpendicularly while experiencing in-plane Lorentz force to converge the beam in one direction and diverge the beam in the other direction. **c**, Experimental setup showing the electrostatic scanner, the single-spaced triplet of quadrupole lenses, and the vacuum chamber in which the samples are mounted, arranged in turn along the α-beam transportation direction.

Supplementary Note 2. Beam spot size determination.

The beam spot sizes are determined by imaging a nickel-grid resolution standard, which was made by using the technique of proton beam writing[2,3]. In our study, we used a focused beam of 1.6 MeV helium ions (α-particles). We scanned the grid with the focused α-beam and detected the energy of the transmitted α-particles, thus a density map of the grid was obtained by calculating the energy-loss of the transmitted α-particles pixel by pixel. This was performed with scanning transmission ion microscopy[4]. Supplementary Fig. 2a shows high-magnification scanning transmission ion microscopy images of the grid.

To determine the beam spot sizes, two representative line-scan profiles were extracted along the horizontal and vertical edges of the grid bars within the boxes as depicted in Supplementary Fig. 2b. The edge-profile data (counts versus line-scan length) are shown in Supplementary Figs. 2c,d, respectively. We then used a modified Gaussian function, see equation (S1), to fit the data. This enabled us to measure the full width at half maximum (FWHM) and therefore determine the beam-spot sizes.

$$Y = A\left[1+\mathrm{erf}\left(2\sqrt{\ln 2}\,\frac{a-X}{f}\right)\right]+B\exp\left[-\ln 16\left(\frac{a-X}{f}\right)^2\right]+C \qquad \text{(S1)},$$

where A, B, and C are unitless fitting parameters, a is a fitting parameter indicating the position of the bar edge, and f represents FWHM. In this case, the unit of a and f is in nm.

The reason we chose to use such a modified Gaussian function is to take into account the effects of enhanced ion-induced electron emission at the edges[5]. The same method was used to measure the nanodiamond sizes and determine the imaging resolution in Fig. 1.

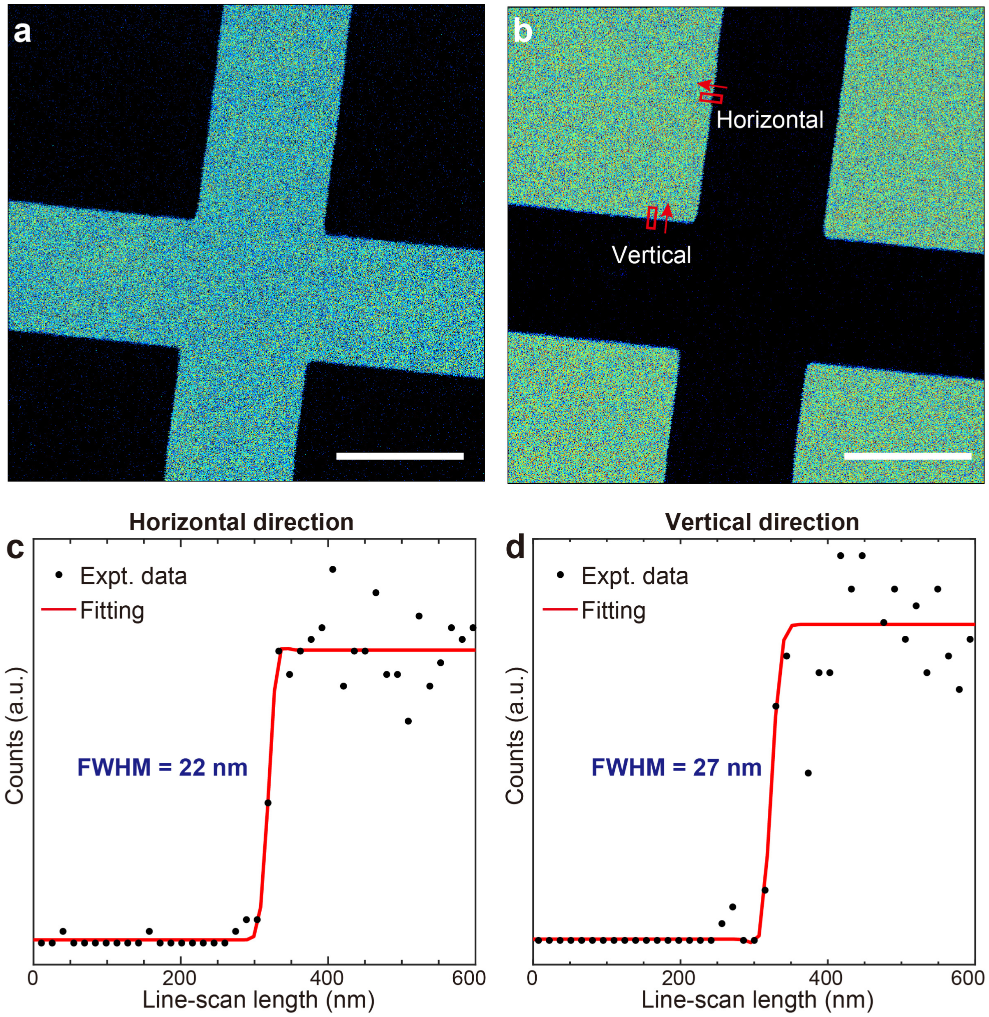

Supplementary Fig. 2 | Beam-spot size measurement of 1.6 MeV focused α-particles. a, Scanning transmission ion microscopy image showing the density map of the grid bars. The scale bar is 4 μm. **b**, Scanning transmission ion microscopy image showing the space between the grid bars. The scale bar is 4 μm. **c**, The corresponding line-scan profile extracted from the counts-data at the region marked in **b** along the horizontal arrow, indicating a horizontal beam-spot size of 22 nm. **d**, The corresponding line-scan profile extracted from the counts-data at the region marked in **b** along the vertical arrow, indicating a vertical beam-spot size of 27 nm.

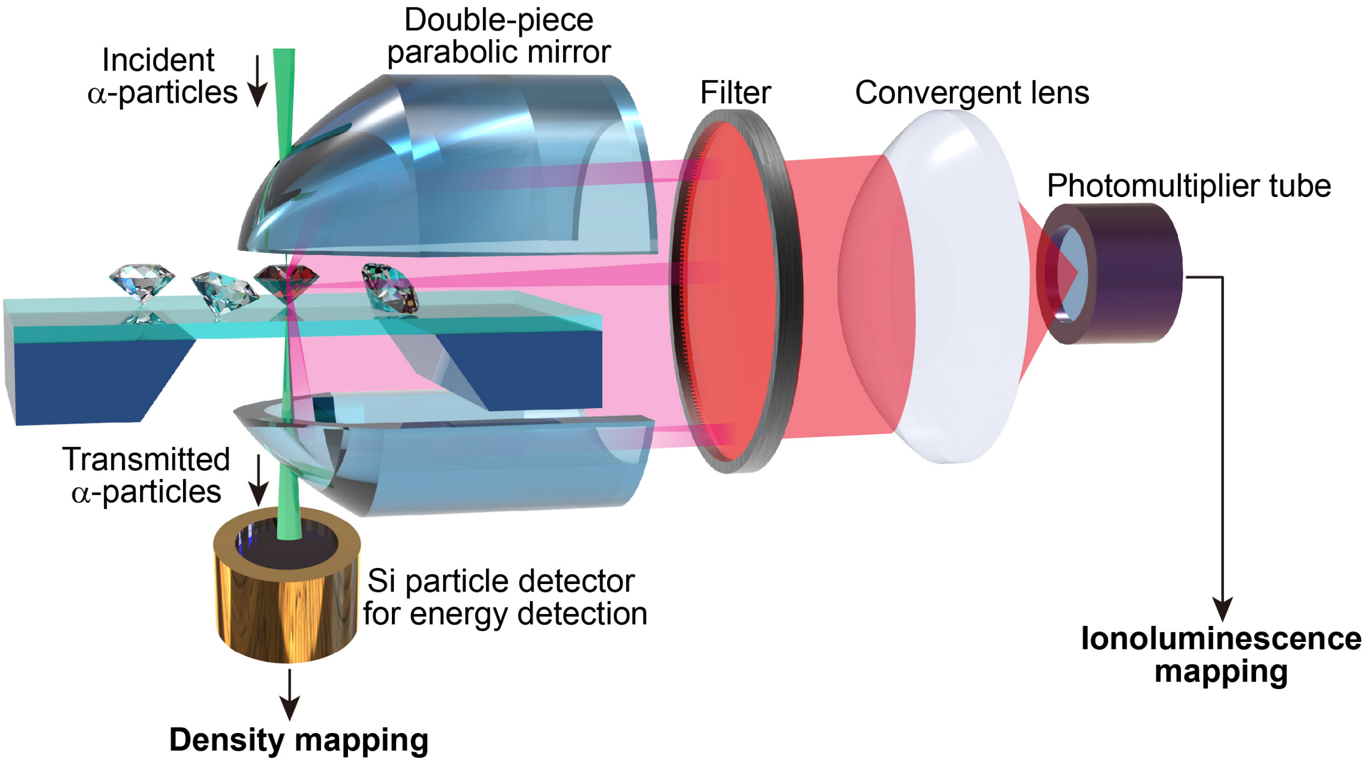

Supplementary Fig. 3 | Schematic illustration of simultaneous density and ionoluminescence mapping. The parabolic mirror, which is made of high-quality aluminum and coated with highly reflective silver at the inner side, is designed to collect the photons emitted from the sample. At the same time, the mirror allows the beam to pass through freely with the design of a through-hole. The scanning beam of focused α-particles pass through the entrance hole in the front piece of the parabolic mirror and interact with the sample. The α-particles lose energy as they pass through the sample, and exit the hole in the rear piece of the mirror. A silicon detector placed downstream of the mirror is used to measure the energy of the transmitted α-particles, and the energy-loss of the α-particles transmitted through the sample can be used to generate a density map of the sample. Simultaneously, the emitted photons from the sample are collected by the mirror and detected with a photomultiplier tube to perform ionoluminescence imaging.

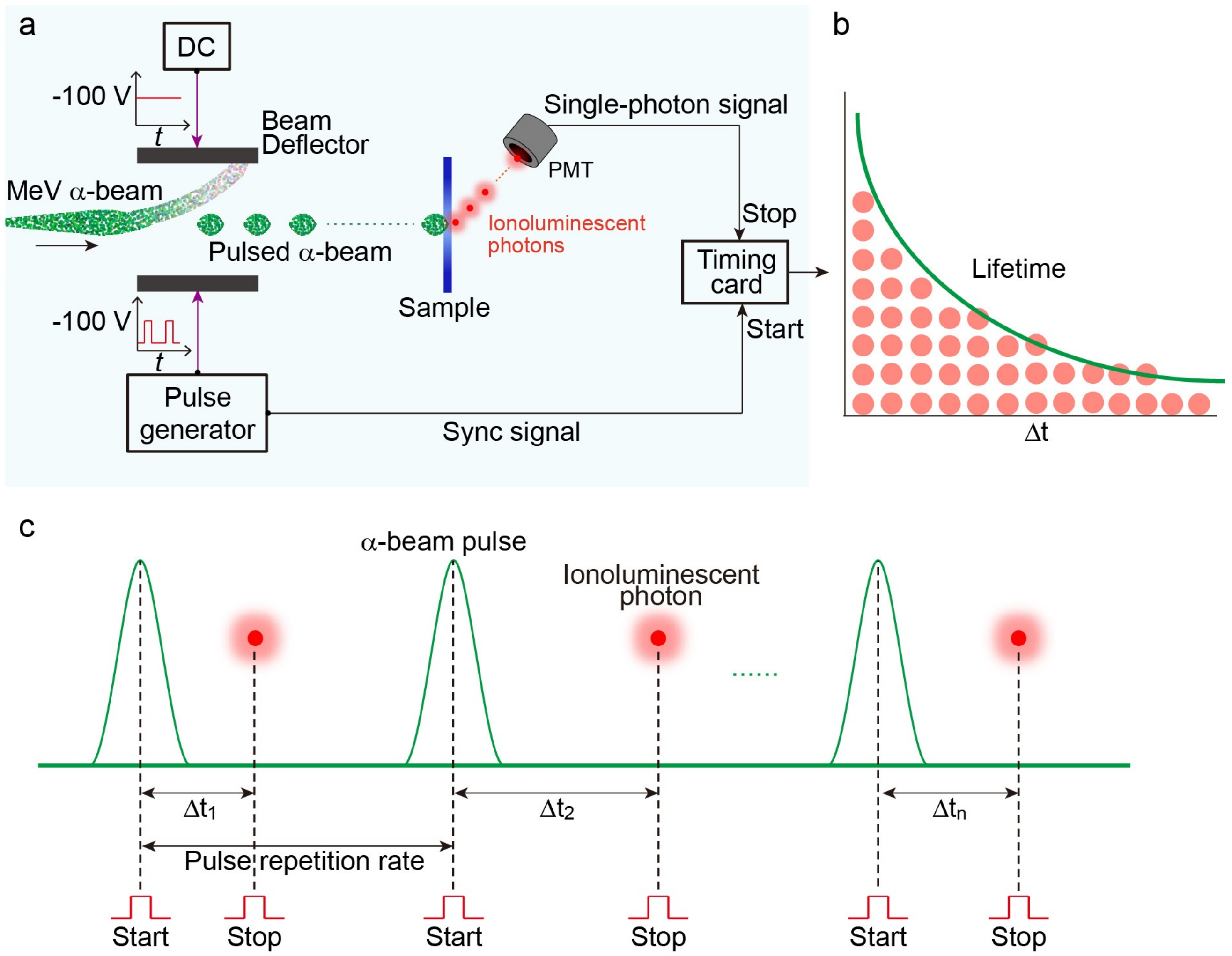

Supplementary Fig. 4 | Time-resolved ionoluminescence. a, Schematic presentation of the time-correlated single-photon counting system we constructed for time-resolved ionoluminescence measurement. Note that a DC power supply (output: -100 V) is used to balance the output voltage of the pulse generator, so that the α-beam is at its 'ON' state (i.e. not deflected) when the sync signal is generated. In this way, we can use the sync signal to trigger the timing card. **b**, Demonstration of the typical exponential-decay result in time-resolved ionoluminescence experiments. A single-photon statistical histogram is formed by registering photon arrivals per time-bin. The width of the time-bin corresponds to the resolution of the timing hardware. **c**, An illustration showing how the histogram in **b** is formed over multiple start-stop cycles.

Supplementary Note 3. Instrumental response function for time-resolved ionoluminescence.

The time response of the pulse generator we employed is 10 ns. When the lifetime of the luminescent samples is not significantly longer than 10 ns, it is important to ascertain the instrumental response function (IRF). To obtain the IRF of our system, we used a fast-decay luminescent material of InGaN quantum-wells as a reference. As shown in Supplementary Fig. 5a, the photoluminescence lifetime of the InGaN quantum wells is 1.3 ns when excited with a 405-nm pulsed laser. We thus expected that quantum-wells are similarly fast for IRF determination in time-resolved ionoluminescece measurement.

InGaN quantum wells exhibit considerable emission upon α-particle excitation, as shown in Supplementary Fig. 5b of the ionoluminescence spectrum. As expected, the ionoluminescence lifetime of the InGaN quantum wells is faster than 10 ns (see Fig. 2). We can therefore use the time-resolved ionoluminescence profile of the InGaN quantum wells to test the IRF of our system. Thereafter, by using the same single-photon collection and detection configuration, we can determine the ionoluminescence lifetimes of materials whose lifetime is longer than 10 ns. The ionolunimescnece lifetime of the NV^0 centers in nanodiamonds was determined in this way.

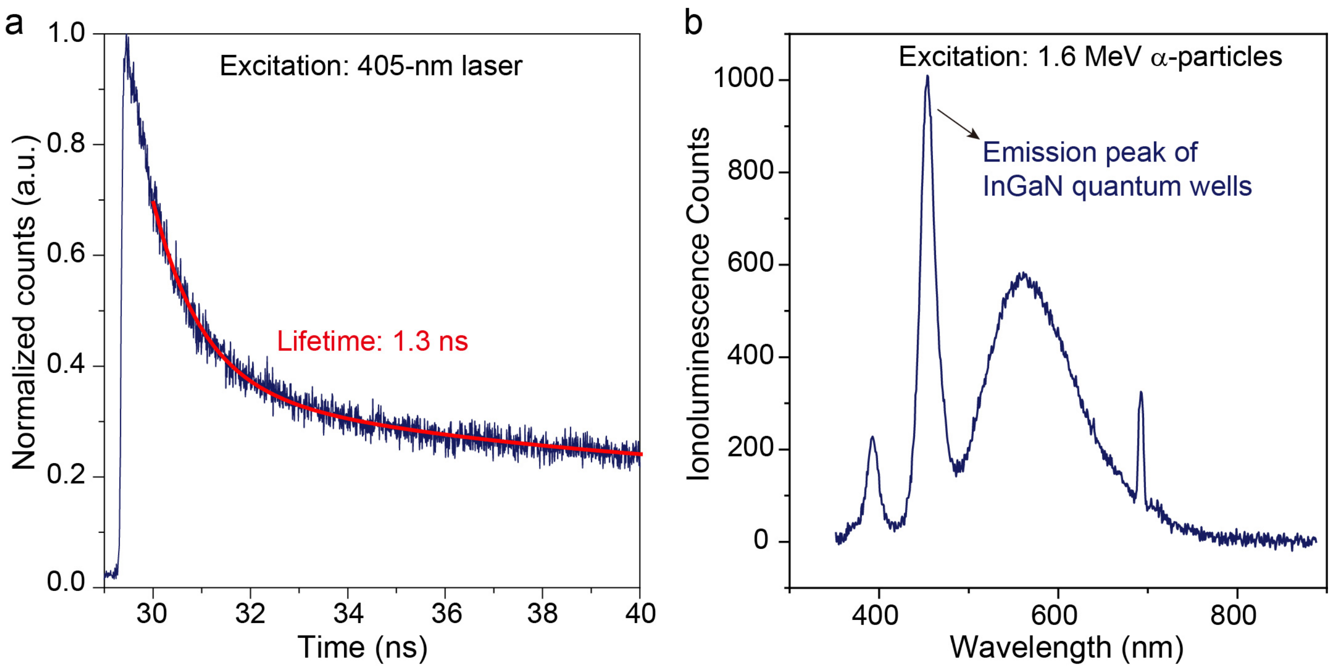

Supplementary Fig. 5 | InGaN quantum-wells for instrumental response function determination. a, Time-resolved photoluminescence measurement of InGaN quantum wells, excited with a pulsed 405-nm laser. **b**, Ionoluminescence spectrum of the InGaN quantum wells, excited with a beam of 1.6 MeV α-particles. Note that the peak centered at 454 nm is the emission from InGaN quantum wells.

Supplementary Note 4. Relative ionoluminescence yield measurement.

To assess the ionoluminescence emission brightness of the fluorescent nanodiamonds under study, we have measured the relative ionoluminescence yield and compared them with several other types of luminescent nanomaterials that are typically employed as probes in biological imaging. These imaging probes include upconversion nanocrystals ($NaYF_4$: Yb/Tm), quantum dots (CdSe/ZnS), and organic dyes (Fluorescein FITC-1907). We prepared each sample onto silicon nitride membranes (100-nm thick) and performed correlative ionoluminescence imaging and scanning transmission ion microscopy of them, by using a focused beam of 1.6 MeV α-particles. Note that scanning transmission ion microscopy images, which showed the density contrast of the samples, were used to determine the energy loss of the α-particles.

The way to calculate the relative ionoluminescence yield is as described in Methods. A scintillation material containing perovskite quantum-dots ($CsPbBr_3$) was used as a reference in the measurements. The perovskite quantum dots were spin-coated onto a piece of quartz glass. The resulting thickness of the perovskite scintillator is about 500 μm with a density of approximately 4.9 g/cm^3. The ionoluminescence spectrum taken from the perovskite scintillator by using 1.6 MeV α-particles is shown in Supplementary Fig. 6. The raw images of nanodiamonds, upconversion nanocrystals, CdSe/ZnS quantum dots, and fluorescein dyes are presented in Supplementary Fig. 7.

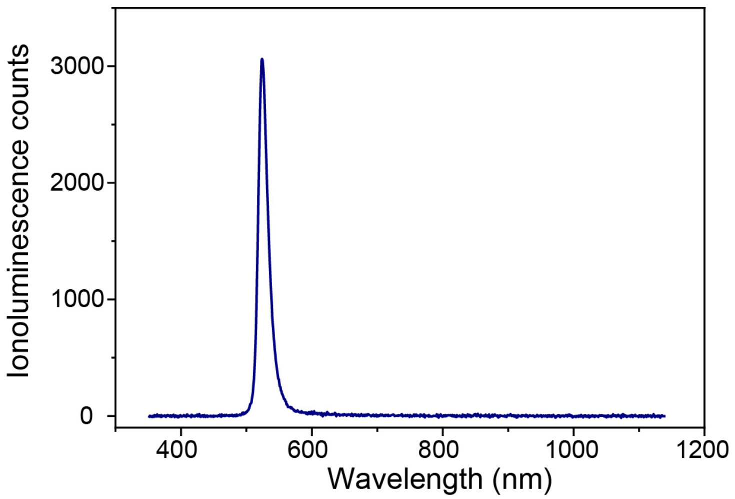

Supplementary Fig. 6 | Ionoluminescence of the $CsPbBr_3$ perovskite quantum-dots scintillator. The spectrum was taken with a beam of 1.6 MeV α-particles.

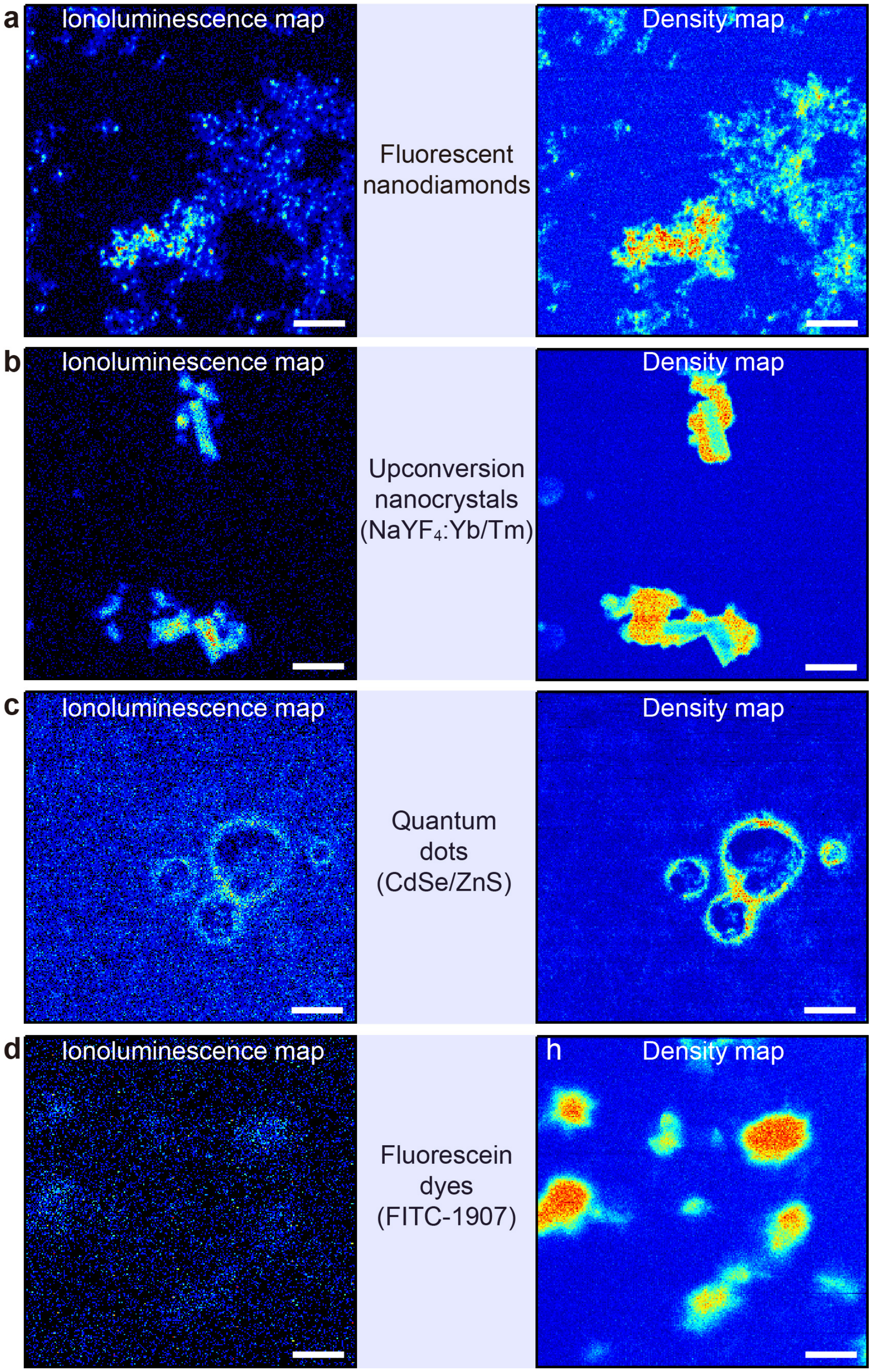

Supplementary Fig. 7 | Raw images of the luminescent nanomaterials under study. a, Fluorescent nanodiamonds. **b**, Upconversion nanocrystals ($NaYF_4$: Yb/Tm). **c**, CdSe/ZnS quantum dots. **d**, Fluorescein dyes (FITC-1907). Note that for each of the samples, the ionoluminescence map and the density map were obtained simultaneously. Scale bars in the images are all 2 μm.

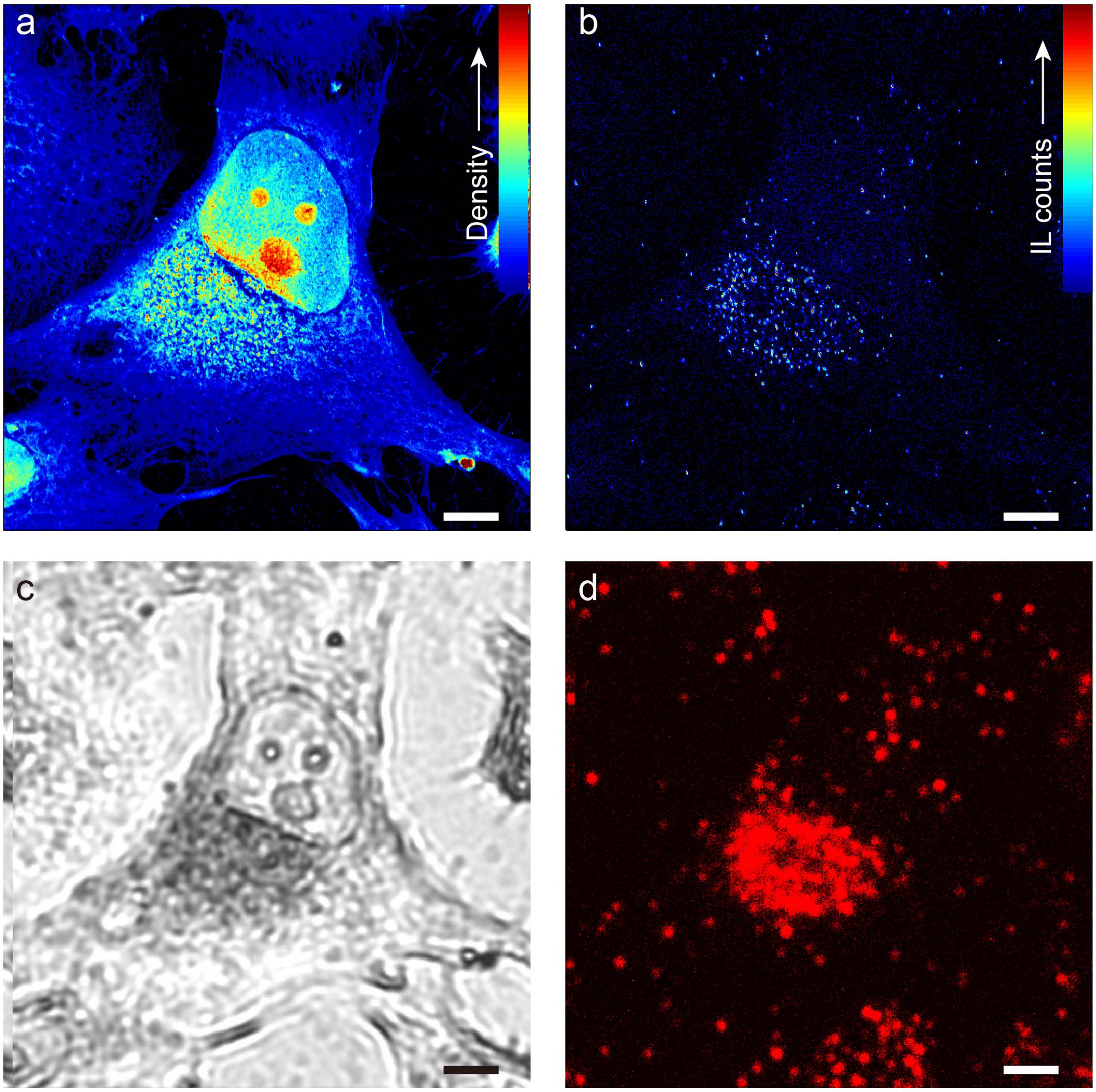

Supplementary Fig. 8 | Single whole HeLa cell imaging. a, Scanning transmission ion microscopy image showing structures of a whole HeLa cell. **b**, Ionoluminescence (IL) image of the same cell showing the emission from nanodiamonds excited by a focused beam of 1.6 MeV α-particles, which illustrates the bio-distribution of nanodiamonds within the whole cell. **c**, Optical image taken by using a 20× objective lens. **d**, Confocal photoluminescence image of the same cell showing nanodiamonds emission excited with a 543-nm helium-neon laser, which indicates a much-reduced resolution compared to the ionoluminescence image, due to the diffraction of light. Scale bars are all 5 μm.

Supplementary Note 5. Possibility of live-cell ion beam imaging.

In our ion beam imaging technique, the cells were dried (by using the technique of critical point drying). Each ionoluminescence image and scanning transmission ion microscopic image were taken and recorded by a 1024 x 1024 pixel array, respectively, with a typical dwell time at each pixel of 1717 μs, at an α-particle count rate of 12000/s. For a typical cellular scanning area of $50 \times 50\ \mu m^2$ and dried cellular thickness of about 2 μm, the corresponding fluence was $8.64 \times 10^{11}\ cm^{-2}$. In this case, the dose for 1.6 MeV alpha particles (the linear energy transfer is about 200 keV/μm) was estimated to be 2.54×10^5 Gy, which is considered much higher than the dose needed (1-10 Gy) for live-cell irradiation in radiotherapy.

For investigations into live cells, this dose is around 4 orders of magnitude above the lethal dose of live cells known to cause cell death. Therefore, the dose needed to get a good contrast for α-particle imaging is probably unrealistic for imaging biological activities in a living cell. This was similarly noted by researchers who work on live cell electron microscopy[6]. However, if real-time imaging of biological activities is not necessarily needed or for imaging of non-biological specimens in the liquid status, our α-beam imaging approach is technically extensible.

The idea is similar to that of electron microscopy in imaging specimens in liquid[7,8]. In our proposal, the ion beam travels in the vacuum before entering the enclosed liquid medium for live-cell imaging. As such, the ion energy loss and beam spot spread are not affected by the air but by the thickness of the cell. For a typical human cancer cell line, for example, the liver cancer cell HepG2, the thickness of the nuclear region can be as thick as about 10 μm, while the cytoplasmic region can be less than 2 μm in thickness. To estimate the spot spread of a 2-MeV proton beam traveling in a live cell, we performed SRIM simulations into liquid water that simulates the cells of a similar density. The simulation results indicate that the beam spot spread after penetrating the first 2 μm water is about 13 nm and is increased to about 169 nm after traveling up to 10 μm in water. Therefore, assuming an initial spot size of 20 nm for a 2-MeV proton beam, we would estimate a sub-40 nm spatial resolution for imaging the cytoplasmic region and sub-200 nm for imaging the nuclear region of the cell, which are supposed to be lower than the optical diffraction limit.

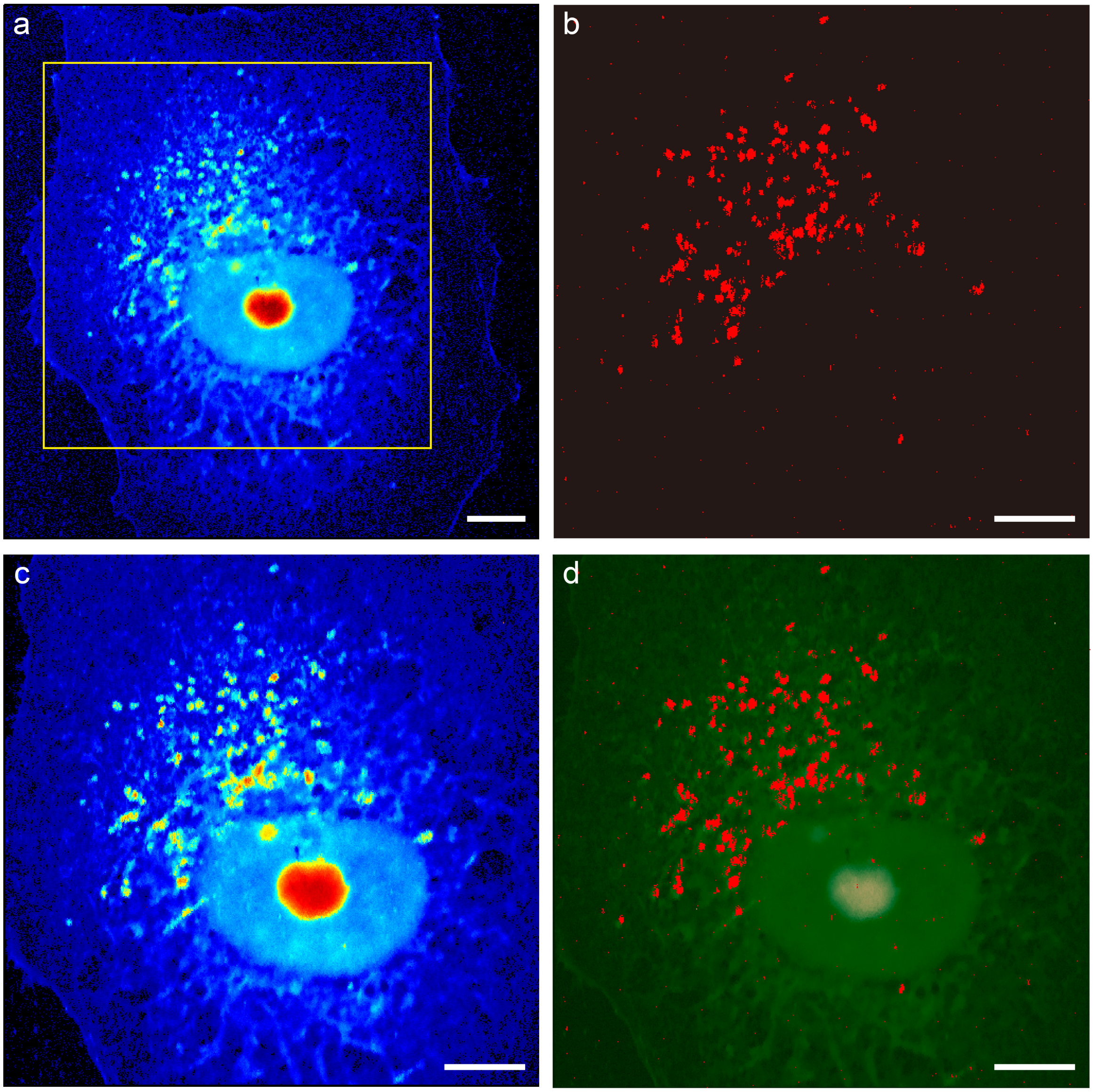

Supplementary Fig. 9 | Single whole HepG2 cell imaging. a, Scanning transmission ion microscopy image showing structures of a whole HepG2 cell. **b**, Ionoluminescence image of the cell area depicted in **a**, showing the emission from nanodiamonds excited by a focused beam of 1.6 MeV α-particles **c**, Scanning transmission ion microscopy image showing structures of the cell area depicted in **a**. **d**, Overlay of the ionoluminescent and structural images presented in **b** and **c**, respectively, which illustrates the bio-distribution of the nanodiamonds within the whole HepG2 cell. Scale bars are all 5 μm.

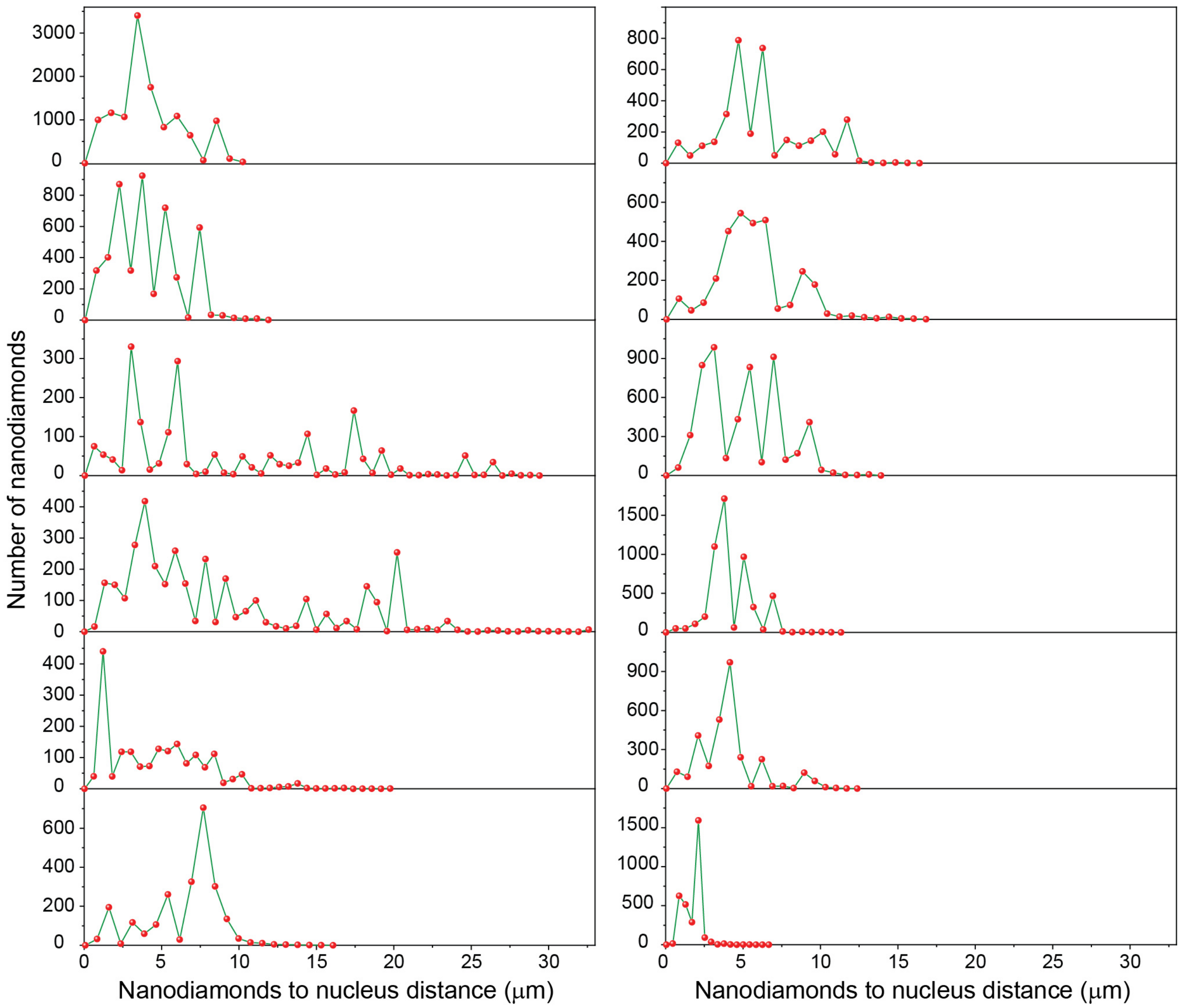

Supplementary Fig. 10 | Quantitative measurement of the nanodiamonds bio-distribution in HepG2 cells. In this experiment, 12 cells were investigated by taking correlative structure and ionoluminescence images using a focused beam of 1.6 MeV α-particles. From these images, the number of nanodiamonds and their localization in the whole cells can be determined. This figure shows the results of the 12 cells by measuring the number of nanodiamonds in terms of their distance to the cell nuclear boundary.

Supplementary Note 6. Simulation considerations based on a clinically relevant dose.

In Monte Carlo simulations by using Geant4-DNA, we simulated 1×10^7 protons to achieve a reasonable accuracy of statistics. Realistically in cell irradiation with protons, a clinically relevant dose is ranged from 1-10 Gy for different cell types and proton energies[9-12]. Here, in our calculations we used a typical dose of 2 Gy for 2-MeV proton irradiation of HepG2 cells. Based on the dose of 2 Gy, we can calculate the number of protons needed for irradiating a HepG2 cell by using equation (S2) which assumes homogeneous irradiation.

$$D = \frac{S}{\rho} \cdot \frac{N}{A} \quad \text{(S2)},$$

where D presents the dose in the unit of grey (Gy) which equals to J/kg, S is the stopping power, ρ is the mass density of the cell which in this case is simulated with water, and N is the number of protons for irradiating a cell area of A. In our study, the typical area of a HepG2 cell is about 2500 μm^2 (see Supplementary Fig. 9), and the mass stopping power (S/ρ) of a 2-MeV proton traveling in liquid water is 158.6 MeV/(g/cm^2) which was obtained by using SRIM simulation[13]. As such, the number of protons needed for a dose of 2 Gy is calculated as 1970.

Next, we can estimate the average number of protons shared by each nanodiamond, based on some experimental values. First, the cellular area that contains the nanodiamonds was estimated to be about 300 μm^2 (see Supplementary Fig. 9). So, on average the number of protons going through this nanodiamond-containing area is $(300/2500) \times 1970 \approx 236$. The mean number of the nanodiamonds internalized by a HepG2 cell was determined to be 4142 from our experiment (Supplementary Table 1). Therefore, the 236 protons would be hitting about $236/4142 \approx 6\%$ of the nanodiamonds, at 1 proton per hit nanodiamond, that is, one nanodiamond will experience the impact of 0.06 protons on average.

Supplementary Table 1. Number of nanodiamonds in individual HepG2 cells.

Cell label	#1	#2	#3	#4	#5	#6	#7	#8	#9	#10	#11	#12
Total number of nanodiamonds	12119	4699	1965	3473	1804	2347	3475	3095	5401	5115	3023	3188
Mean number of nanodiamonds	4142											
Standard deviation	2764											

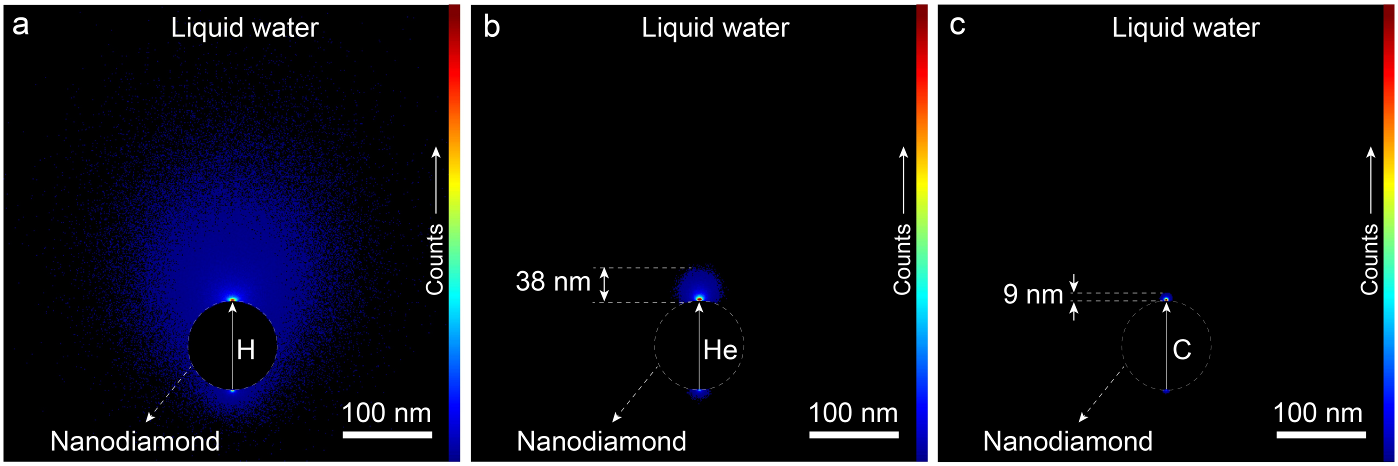

Supplementary Fig. 11 | Range distributions of the secondary electrons derived from the nanodiamond with the impact by different ion species. a, Image showing the secondary electrons derived from proton-nanodiamond interaction can travel up to a few 100 nm in liquid water. **b**, Image showing the secondary electrons derived from helium ion-nanodiamond interaction can travel about 38 nm in liquid water. **c**, Image showing the secondary electrons derived from carbon ion-nanodiamond interaction can only travel less than 10 nm in liquid water. All simulations were performed with Geant4-DNA. The energy and the number of ions used for each simulation were 2 MeV and 1×10^5, respectively. Note that we assumed the ions (protons, helium ions, and carbon ions) travel only within the nanodiamond.

Supplementary Note 7. Measurement of the size of nanodiamonds.

To measure the size of the nanodiamonds, we performed scanning electron microscopic (SEM) analysis of the nanodiamond sample prepared onto a 100-nm-thick SiN membrane substrate. To eliminate the charging effect during SEM imaging, the sample was coated with a 5-nm layer of platinum by using a JEOL JFO-1600 Auto Fine Coater. We took multiple SEM images of the nanodiamonds at different regions of the sample. Supplementary Fig. 12a shows a representative one of them. These images were then analyzed using ImageJ software to measure the sizes of individual nanodiamonds. The result is shown in Supplementary Fig. 12b to form a histogram of the size distribution of the nanodiamonds.

The function of the fitted curve of the histogram is presented by equation (S3), where N is the frequency of occurrence of the nanodiamonds and D is the size of the nanodiamonds.

$$N = 3.0 + 66.9\exp\left[-\frac{1}{2}\left(\frac{D-86.8}{30.4}\right)^2\right] \tag{S3}$$

The fitted result shows that the mean size of the nanodiamonds is 86.8 nm and the standard deviation is 30.4 nm. The large standard deviation is a result of the considerable size variation of the nanodiamonds from about 20 nm to around 200 nm.

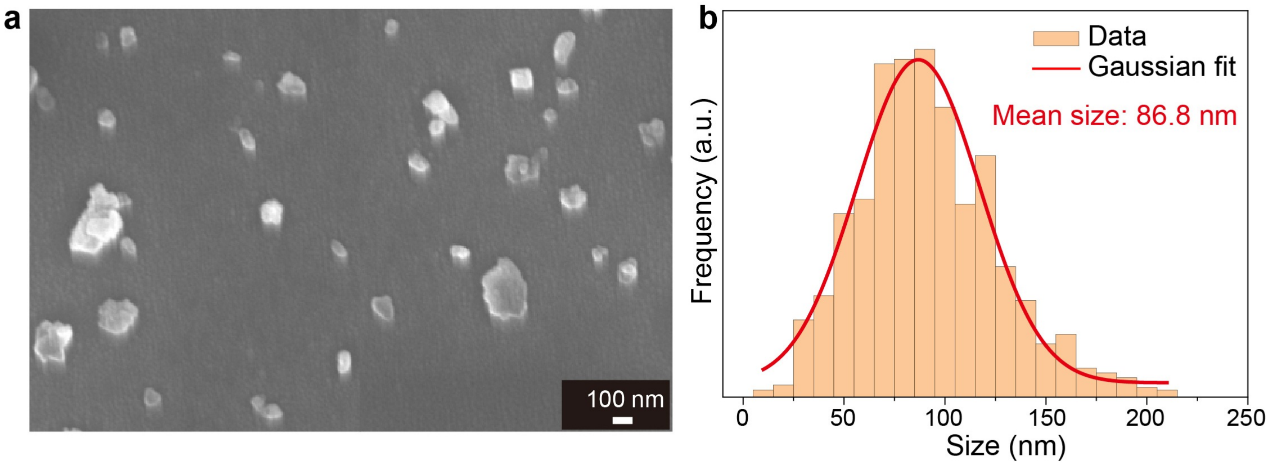

Supplementary Fig. 12 | Scanning electron microscopic analysis. a, A typical scanning electron microscopic image of the nanodiamonds. **b**, Size distribution histogram of the nanodiamonds, showing a mean size of 86.8 nm.

Supplementary Note 8. Image segmentation for nanodiamond counting in cells.

The image segmentation aims to identify individual nanodiamonds and their clusters in the ionoluminescence image and differentiate them from the background. The algorithm chain of performing image segmentation is shown in Supplementary Fig. 13. First, for ionoluminescence images with weak signals of photon counts, random Poisson noise will mask the image. There can be zero-value pixels existing within the image area containing nanodiamonds, which makes the nanodiamonds appear to be discontinuous. The method to deal with this issue is to use a low-pass filter. Second, the ionoluminescence image is converted to a binary image to identify the higher-intensity pixels from the black background, accomplished by using a local thresholding algorithm. Third, it should be noted that the thresholding step will identify the random noise as well. However, the noise signal is unlikely to form a connected area of white pixels due to its random nature. We can therefore impose a threshold area such that all connected areas below this threshold are not regarded as areas containing nanodiamonds. Finally, we can obtain a binary image containing only the individual nanodiamonds and their clusters.

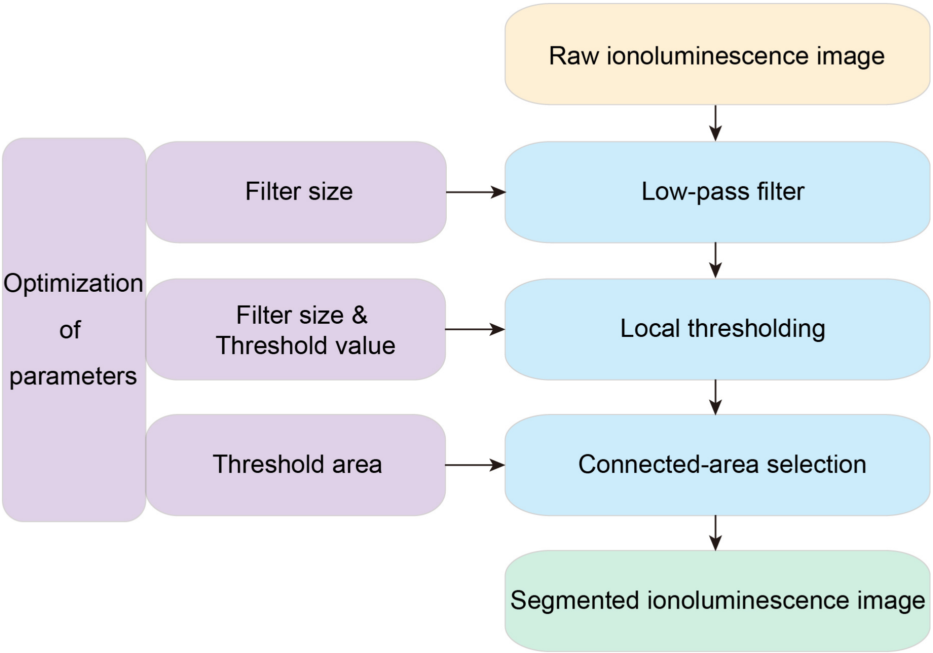

Supplementary Fig. 13 | Algorithm chain of ionoluminescence image segmentation. The optimization of the filter size aims to reduce the noise but not affect the signal significantly. The efficacy of the local thresholding algorithm depends on the filter size and the threshold value.

Supplementary Table 2. Travel-range simulations of 7 chemical species by using Geant4-DNA through radiation chemistry modeling[14,15]. The values are calculated based on an average impact by 0.06 protons as described in Supplementary Note 6. Note that e_{aq}^- represents hydrated electrons.

Chemical species	H_2O_2	H_2	OH^-	H•	e_{aq}^-	•OH	H_3O^+
Total counts for travel range > 457 nm (reaching cell nuclues)	0.003	0.004	0.007	0.008	0.015	0.015	0.027
Total counts for travel range ≤ 457 nm (in cell cytoplasm)	0.218	0.150	0.383	0.361	0.999	1.157	0.797

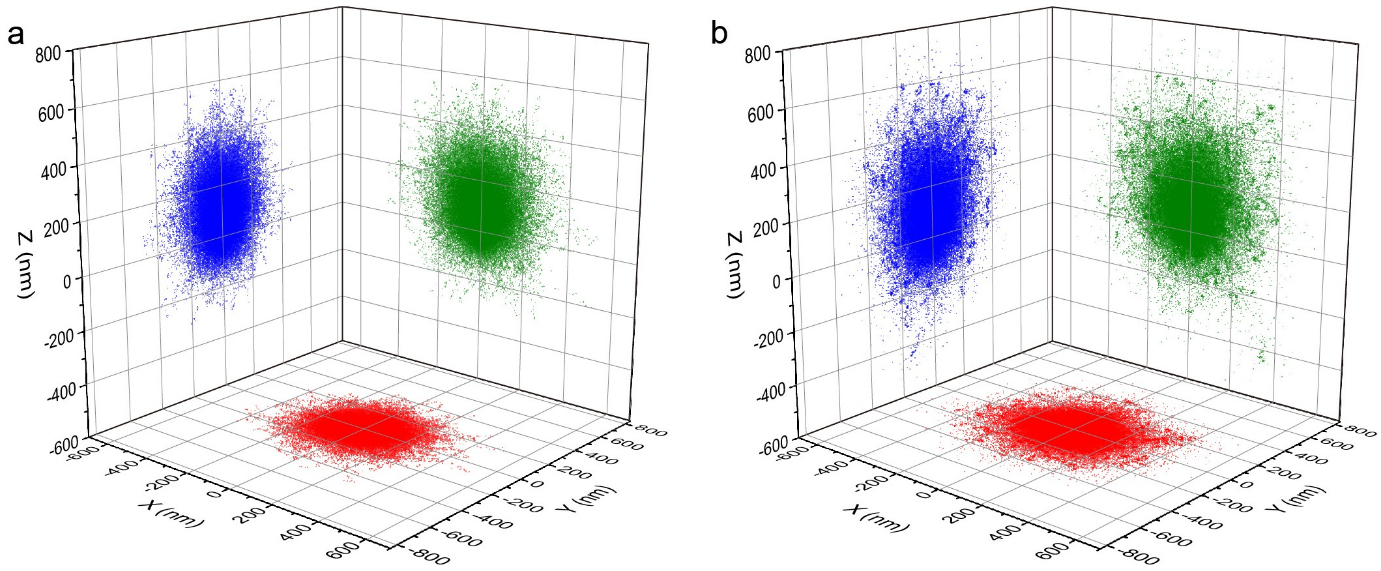

Supplementary Fig. 14 | Geant4-DNA Monte Carlo simulations. a, Three-dimensional ranges of the induced secondary electrons. **b**, Three-dimensional ranges of the induced hydroxyl radicals. Note that each dot (shown in red, green, and blue) at the projection planes represents a stop-site of a secondary electron (in **a)** or a hydroxyl radical (in **b)**. The primary 2-MeV proton beam is assumed to travel in parallel with the Z-axis within a nanodiamond and stop at the point (0, 0, 0) which is on the surface of the nanodiamond.

Supplementary Note 9. Foci counting in 3D confocal microscopic images.

Three-dimensional confocal microscopic images of proton-irradiated or control cells were obtained through z-stacking of 2D image slices. To count the γH2AX or 53BP1 foci in these cells, a custom code was developed to perform image processing and subsequent foci counting automatically. First, the 2D image slices were processed to define the nuclei of the cells. The nucleus region was then used as a mask to remove the noise outside the defined region in the foci images. Secondly, the fluorescence intensity of all 2D image slices at each imaging pixel was compared to determine a maximum value. Each maximum value was then assigned to the corresponding pixel. As a result, a 2D projection image was formed. This 2D image was subsequently corrected for uneven illumination and some large artefacts were removed. The foci were then obtained as the foreground in the processed 2D projection image by applying a method of automatic multilevel thresholding[16]. As a result, a binary image was formed. In this binary image, each connected region was defined as a single object. The number of foci was thus determined by counting the number of connected regions in the resulting binary image.

Supplementary References

1. Watt, F. et al. The Singapore high resolution single cell imaging facility. *Nucl. Instrum. Methods Phys. Res. B* **269,** 2168-2174 (2011).
2. Watt, F., Breese, M. B. H., Bettiol, A. A. & van Kan, J. A. Proton beam writing. *Mater. Today* **10,** 20-29 (2007).
3. Zhang, F., van Kan, J. A., Chiam, S. Y. & Watt, F. Fabrication of free standing resolution standards using proton beam writing. *Nucl. Instrum. Methods Phys. Res. B* **260,** 474-478 (2007).
4. Chen, X. et al. High-resolution 3D imaging and quantification of gold nanoparticles in a whole cell using scanning transmission ion microscopy. *Biophys. J.* **104,** 1419-1425 (2013).
5. Udalagama, C. N. B. et al. An automatic beam focusing system for MeV protons. *Nucl. Instrum. Methods Phys. Res. B* **231,** 389-393 (2005).
6. de Jonge, N. & Peckys, D. B. Live cell electron microscopy is probably impossible. *ACS Nano* **10**, 9061-9063 (2016).
7. Park, J. et al. Direct observation of wet biological samples by graphene liquid cell transmission electron microscopy. *Nano Lett.* **15**, 4737-4744 (2015).
8. Keskin, S., Kunnas, P. & de Jonge, N. Liquid-phase electron microscopy with controllable liquid thickness. *Nano Lett.* **19**, 4608-4613 (2019).
9. Auer, S. et al. Survival of tumor cells after proton irradiation with ultra-high dose rates. *Radiat. Oncol.* **6,** 139 (2011).
10. Alan Mitteer, R. et al. Proton beam radiation induces DNA damage and cell apoptosis in glioma stem cells through reactive oxygen species. *Sci. Rep.* **5,** 13961 (2015).
11. Shahmohammadi Beni, M., Krstic, D., Nikezic, D. & Yu, K. N. Medium-thickness-dependent proton dosimetry for radiobiological experiments. *Sci. Rep.* **9,** 11577 (2019).
12. Szymonowicz, K. et al. Proton irradiation increases the necessity for homologous recombination repair along with the indispensability of non-homologous end joining. *Cells* **9,** 889 (2020).
13. Ziegler, J. F., Ziegler, M. D. & Biersack, J. P. SRIM – The stopping and range of ions in matter (2010). *Nucl. Instrum. Methods Phys. Res. B* **268,** 1818-1823 (2010).
14. Karamitros, M., Incerti, S. & Mantero, A. Modeling radiation chemistry in the Geant4 toolkit. *Progr. Nucl. Sci. Technol.* **2,** 503-508 (2011).
15. Bernal, M. A. et al. Track structure modeling in liquid water: A review of the Geant4-DNA very low energy extension of the Geant4 Monte Carlo simulation toolkit. *Phys. Med.* **31,** 861-874 (2015).
16. Yen, J.-C., Chang, F. & Chang, S. A new criterion for automatic multilevel thresholding. *IEEE Trans. Image Process.* **4**, 370-378 (1995).
