# Supplementary material for: Quantifying nanodiamonds biodistribution in whole cells with correlative iono-nanoscopy
Source: Nat Commun. 2021 Aug 2;12:4657. doi: 10.1038/s41467-021-25004-9 (PMC8329174; doi:10.1038/s41467-021-25004-9)
Supplement: Supplementary file 2 — Reporting Summary [file 41467_2021_25004_MOESM2_ESM.pdf]

nature research

Corresponding author(s): Andrew A. Bettiol, Frank Watt

Last updated by author(s): Jul 5, 2021

Reporting Summary

Nature Research wishes to improve the reproducibility of the work that we publish. This form provides structure for consistency and transparency in reporting. For further information on Nature Research policies, see our Editorial Policies and the Editorial Policy Checklist.

Statistics

For all statistical analyses, confirm that the following items are present in the figure legend, table legend, main text, or Methods section.

n/a	Confirmed	
☐	☒	The exact sample size (n) for each experimental group/condition, given as a discrete number and unit of measurement
☐	☒	A statement on whether measurements were taken from distinct samples or whether the same sample was measured repeatedly
☐	☒	The statistical test(s) used AND whether they are one- or two-sided<br>*Only common tests should be described solely by name; describe more complex techniques in the Methods section.*
☒	☐	A description of all covariates tested
☒	☐	A description of any assumptions or corrections, such as tests of normality and adjustment for multiple comparisons
☐	☒	A full description of the statistical parameters including central tendency (e.g. means) or other basic estimates (e.g. regression coefficient) AND variation (e.g. standard deviation) or associated estimates of uncertainty (e.g. confidence intervals)
☒	☐	For null hypothesis testing, the test statistic (e.g. F, t, r) with confidence intervals, effect sizes, degrees of freedom and P value noted<br>*Give P values as exact values whenever suitable.*
☒	☐	For Bayesian analysis, information on the choice of priors and Markov chain Monte Carlo settings
☒	☐	For hierarchical and complex designs, identification of the appropriate level for tests and full reporting of outcomes
☒	☐	Estimates of effect sizes (e.g. Cohen's d, Pearson's r), indicating how they were calculated

Our web collection on statistics for biologists contains articles on many of the points above.

Software and code

Policy information about availability of computer code

Data collection	Ionoluminescence image data were collected on a custom-built ion-beam microscope which is described in Methods. The ion-beam microscope is controlled by a custom-made controlling software named IonDAQ (version 1.0, Ref: Bettiol, A. A. et al. Nucl. Instrum. Methods Phys. Res. B 267, 2069-2072, 2009). Ionoluminescence spectroscopic data were collected with the SpectraSuite software (Ocean Optics). Ionoluminescence lifetime data were collected with a custom-built time-resolved ionoluminescence system, using the software TimeHarp 260 v3.1 (PicoQuant). The Monte Carlo simulation data were generated with the Geant4-DNA package which is an extension to the Geant4 (version 10.5) Monte Carlo toolkit. The confocal microscopic images of cells were taken using an LSM 880 laser scanning confocal microscope (Carl Zeiss).
Data analysis	The ion-beam image processing was performed using a custom software named ionDAQ-lite (Ref: Bettiol, A. A. et al. Nucl. Instrum. Methods Phys. Res. B 267, 2069-2072, 2009). ImageJ 1.48v was used to generate line profiles from images. Ionoluminescence spectroscopic data, lifetime data, and curve fitting were analysed with Origin 2018b. MATLAB R2018b (MathWorks) was used for 3D visualization of the cells. Algorithms of image segmentation and nanodiamond counting were realized by MATLAB R2018b. Geant4-DNA simulation data was also analysed with MATLAB. The confocal microscopic images of cells were processed and the foci number was thus counted by coding with Python 3.7. The custom codes for image processing and foci counting in confocal images of cells are publicly accessible at https://github.com/CIBA-Physics-NUS/FociApp.

For manuscripts utilizing custom algorithms or software that are central to the research but not yet described in published literature, software must be made available to editors and reviewers. We strongly encourage code deposition in a community repository (e.g. GitHub). See the Nature Research guidelines for submitting code & software for further information.

Data

Policy information about availability of data

All manuscripts must include a data availability statement. This statement should provide the following information, where applicable:
- Accession codes, unique identifiers, or web links for publicly available datasets
- A list of figures that have associated raw data
- A description of any restrictions on data availability

The datasets that support the findings of this study have been deposited in the Zenodo repository under a Creative Commons Attribution 4.0 International License (Open Access) at http://doi.org/10.5281/zenodo.5068754.

Field-specific reporting

Please select the one below that is the best fit for your research. If you are not sure, read the appropriate sections before making your selection.

[x] Life sciences   [ ] Behavioural & social sciences   [ ] Ecological, evolutionary & environmental sciences

For a reference copy of the document with all sections, see nature.com/documents/nr-reporting-summary-flat.pdf

Life sciences study design

All studies must disclose on these points even when the disclosure is negative.

Sample size	Sample sizes are indicated in this manuscript and supplementary information. In previous validation experiments, we were able to reliably quantify differences in DNA lesions in response to the doses used when the indicated number of cells were analysed (unpublished data). Based on these data, and using $\alpha = 0.05$, $\beta = 0.2$, a sample size of 10 was chosen that allows a minimal detectable effect of 10%.
Data exclusions	No data were excluded from the analyses.
Replication	Replication was performed as indicated in the Methods section. All attempts at replication were successful.
Randomization	Treatment conditions were assigned randomly to cells grown in parallel for each experimental repeat.
Blinding	Because nanodiamonds are visible by fluorescence, operators could not be blinded regarding presence of nanodiamonds during image analysis. To avoid operator bias, all critical steps in foci quantification (image processing, identification and enumeration of foci) were carried out using automated pipeline, excluding any human input. During image acquisition, cells were selected randomly from prepared samples.

Reporting for specific materials, systems and methods

We require information from authors about some types of materials, experimental systems and methods used in many studies. Here, indicate whether each material, system or method listed is relevant to your study. If you are not sure if a list item applies to your research, read the appropriate section before selecting a response.

Materials & experimental systems

n/a	Involved in the study	
[ ]	[x]	Antibodies
[ ]	[x]	Eukaryotic cell lines
[x]	[ ]	Palaeontology and archaeology
[x]	[ ]	Animals and other organisms
[x]	[ ]	Human research participants
[x]	[ ]	Clinical data
[x]	[ ]	Dual use research of concern

Methods

n/a	Involved in the study	
[x]	[ ]	ChIP-seq
[x]	[ ]	Flow cytometry
[x]	[ ]	MRI-based neuroimaging

Antibodies

Antibodies used	γH2AX (Millipore Cat# 05-636-I, RRID:AB_2755003)<br>53BP1 (Novus Cat# NB100-304, RRID:AB_10003037)<br>Goat anti-Mouse IgG Alexa Fluor 568 (Thermo Fisher Scientific Cat# A-11004, RRID:AB_2534072)<br>Goat anti-Rabbit IgG Alexa Fluor 488 (Thermo Fisher Scientific Cat# A-11070, RRID:AB_2534114)<br>Dilution: 1:500
Validation	γH2AX - species mouse. Well-published antibody used extensively for the detection of Ser139 phosphorylated H2AX. Validated by analysis of treated and untreated cells (immunofluorescence; this manuscript and Millipore)<br>53BP1 - species rabbit. Well-published antibody used extensively for the detection of 53BP1. Validated by analysis of treated and

untreated cells (immunofluorescence; this manuscript and Novus), and 53BP1 knockout in cells (immunofluorescence; Novus)

Eukaryotic cell lines

Policy information about cell lines

Cell line source(s)	HeLa and HepG2 were from ATCC.
Authentication	Cell lines were not authenticated independently.
Mycoplasma contamination	Cell lines were tested negative for mycoplasma.
Commonly misidentified lines (See ICLAC register)	Not used.
